# Opposing Activities of DRM and MES-4 Tune Gene Expression and X-Chromosome Repression in *Caenorhabditis elegans* Germ Cells

**DOI:** 10.1534/g3.113.007849

**Published:** 2013-11-26

**Authors:** Tomoko M. Tabuchi, Andreas Rechtsteiner, Susan Strome, Kirsten A. Hagstrom

**Affiliations:** * Department of Molecular Medicine, University of Massachusetts Medical School, Worcester, Massachusetts 01605; † Department of Molecular, Cell, and Developmental Biology, University of California Santa Cruz, Santa Cruz, California 95064

**Keywords:** gene regulation, development, chromatin, germ cells, X chromosome

## Abstract

During animal development, gene transcription is tuned to tissue-appropriate levels. Here we uncover antagonistic regulation of transcript levels in the germline of *Caenorhabditis elegans* hermaphrodites. The histone methyltransferase MES-4 (Maternal Effect Sterile-4) marks genes expressed in the germline with methylated lysine on histone H3 (H3K36me) and promotes their transcription; MES-4 also represses genes normally expressed in somatic cells and genes on the X chromosome. The DRM transcription factor complex, named for its Dp/E2F, Retinoblastoma-like, and MuvB subunits, affects germline gene expression and prevents excessive repression of X-chromosome genes. Using genome-scale analyses of germline tissue, we show that common germline-expressed genes are activated by MES-4 and repressed by DRM, and that MES-4 and DRM co-bind many germline-expressed genes. Reciprocally, MES-4 represses and DRM activates a set of autosomal soma-expressed genes and overall X-chromosome gene expression. Mutations in *mes-4* and the DRM subunit *lin-54* oppositely skew the transcript levels of their common targets and cause sterility. A double mutant restores target gene transcript levels closer to wild type, and the concomitant loss of *lin-54* suppresses the severe germline proliferation defect observed in *mes-4* single mutants. Together, “yin-yang” regulation by MES-4 and DRM ensures transcript levels appropriate for germ-cell function, elicits robust but not excessive dampening of X-chromosome-wide transcription, and may poise genes for future expression changes. Our study reveals that conserved transcriptional regulators implicated in development and cancer counteract each other to fine-tune transcript dosage.

Proper development requires that genes be expressed at appropriate levels in appropriate tissues. Developmental gene regulation often is viewed as a series of all-or-none switches that turn genes on or off to promote cell identity and function. However, a gene that is “on” may only be expressed at moderate levels. Similarly, a gene that is “off” may not be completely or irreversibly inactivated but may instead be expressed at very low levels and poised for reactivation. Such fine-tuning is particularly important for genes for which a relatively small degree of transcriptional variability may have a profound influence on cell identity or function. For example, transcription of Oct3/4, which is critical for self-renewal, is precisely regulated in embryonic stem cells; either too much or too little Oct3/4 expression leads to differentiation (Niwa *et al.* 2000). How the transcriptional regulatory machinery precisely controls and maintains proper transcript levels is not well understood. In some cases, tuning is achieved through the combined action of factors that activate and factors that repress transcription (Reynolds *et al.* 2013). In this study, we investigated gene expression regulation in the germ cells of *Caenorhabditis elegans* and uncovered a system of transcriptional fine-tuning by antagonistic transcriptional regulators. This transcriptional fine-tuning system acts on sets of autosomal genes and on the X chromosomes and is essential for germ-cell development.

Germ cells give rise to gametes and the next generation of an organism. To serve this critical role, germ cells must express genes required for germline functions and silence genes that might interfere with germline development, including genes associated with somatic development. Key regulators of the transcriptional program in *C. elegans* germ cells are the MES histone methyltransferases (Capowski *et al.* 1991). MES-4 methylates histone H3 on lysine 36 (H3K36me), a mark associated with active gene expression (Bender *et al.* 2006; Rechtsteiner *et al.* 2010). MES-2, MES-3, and MES-6 form the worm version of polycomb repressive complex 2 and generate H3K27me3, which leads to gene repression (Bender *et al.* 2004; Ketel *et al.* 2005; Pengelly *et al.* 2013; Xu *et al.* 2001). Together, the MES proteins define domains of germline-expressed genes marked with MES-4 and H3K36me and mutually exclusive domains of germline-repressed genes marked with H3K27me3 (Gaydos *et al.* 2012). Loss of MES-4 or MES-2/3/6 results in down-regulation of germline-expressed genes and ectopic up-regulation of somatically expressed genes (Gaydos *et al.* 2012). These patterns of misexpression are thought to underlie the maternal-effect sterile phenotype displayed by mutants: worms that inherit *mes(+)* product from their mothers develop into fertile adults, whereas worms that do not inherit maternal *mes(+)* product develop into sterile adults (Capowski *et al.* 1991). Thus, the MES proteins cooperate to promote development of healthy germ cells by activating germline genes and repressing somatic genes.

Another feature of gene regulation in *C. elegans* hermaphrodite germ cells is the significant dampening of transcription from the X chromosomes. Somatic cells reduce X-linked gene expression by approximately twofold in XX worms (hermaphrodites) to match expression in XO worms (males) through a process called X-chromosome dosage compensation (Meyer 2010). Germ cells instead exhibit near-complete silencing of the single X in males and partial silencing of both Xs in hermaphrodites (Bean *et al.* 2004; Kelly *et al.* 2002; Strome and Kelly 2006). MES proteins serve pivotal roles in X-chromosome regulation in the germ cells of hermaphrodites. The MES-2/3/6 complex concentrates repressive H3K27me3 on the X chromosomes (Bender *et al.* 2004; Gaydos *et al.* 2012). MES-4 and H3K36me, which are concentrated on the autosomes, antagonize methylation of H3K27 and help focus MES-2/3/6-generated H3K27me3 on the X chromosomes (Bender *et al.* 2004, 2006; Fong *et al.* 2002; Gaydos *et al.* 2012). Loss of MES-4 or MES-2/3/6 results in up-regulation of genes on the X chromosome (Bender *et al.* 2006; Gaydos *et al.* 2012). The sensitivity of the maternal-effect sterile mutant phenotype to X-chromosome dosage (Garvin *et al.* 1998) suggests that up-regulation of X-linked genes contributes to sterility and thus that repression of genes on the X is crucial for normal germline development.

A recent study implicated another player, the multiprotein DRM complex, in germline X-chromosome regulation and showed that DRM loss affects the X in an opposite manner to the MES proteins (Tabuchi *et al.* 2011). DRM is a conserved transcription factor complex that includes a retinoblastoma-related pocket protein (LIN-35), an E2F/DP heterodimer (EFL-1/DPL-1), and the Multi-vulva class B core subunits (LIN-9, LIN-37, LIN-52, LIN-53, and LIN-54) (Harrison *et al.* 2006; Sadasivam and Decaprio 2013; van den Heuvel and Dyson 2008). *C. elegans* DRM and its homologs in other species regulate genes involved in cell cycle and development, and its dysfunction is linked to sterility, developmental defects, and cancer (*e.g.*, Chi and Reinke 2006; Dimova *et al.* 2003; Georlette *et al.* 2007; Korenjak *et al.* 2004; Kudron *et al.* 2013; Litovchick *et al.* 2007; Reichert *et al.* 2010; Sadasivam and Decaprio 2013; Tabuchi *et al.* 2011; Thomas *et al.* 2003). In *C. elegans* germ cells, DRM predominantly localizes to autosomes, yet loss of the DRM subunit LIN-54 leads to excessive repression of X-linked genes (Tabuchi *et al.* 2011). Autosomally concentrated LIN-54 affecting the expression of genes on the X is reminiscent of MES-4. Although DRM and MES-4 share the unique feature of acting on the X from a distance, it was not previously known whether DRM and MES-4 oppositely influence the same set of X-linked genes, and if they antagonistically regulate genes on the autosomes.

In this work, we show that MES-4 and DRM oppositely regulate a common set of genes to maintain proper transcript dosages for germ cells. We found that DRM counteracts activation of germline genes and repression of somatic genes and X-linked genes by MES-4. Loss of either factor oppositely skewed transcript levels of those genes, whereas loss of both restored their levels closer to wild type. Moreover, the maternal-effect sterile phenotype of *mes-4* mutants was ameliorated by concomitant loss of DRM, highlighting the importance of the oppositely-acting gene regulatory activities of MES-4 and DRM for the development of germ cells. Such opposing regulation was particularly striking for genes located on the X chromosome, illustrating how the X chromosomes in *C. elegans* germ cells are not silenced but tuned to low levels of transcription. We propose that the combined action of MES-4 and DRM prevents transcripts from deviating toward excessive or insufficient levels incompatible with germ-cell function, achieves significant but not complete dampening of X chromosome-wide transcription, and perhaps lowers the barrier to future gene expression changes in the soma. This work illustrates how the action of antagonistic transcriptional regulators on common target genes can control the precise levels of transcripts in a tissue during development.

## Materials and Methods

### Strains

All strains were cultured at 20°, using standard methods. The following strains were used: N2 (Bristol) as wild type, *lin-54(n3423) IV/nT1[qIS51]* (IV;V) and *lin-54(n2990) IV/nT1[qIS51]* (IV;V), *mes-4(ok2326)* V*/nT1[qIs51]* (IV;V), *dpy-11(e224) mes-4(bn23) unc-76(e911) V/*D*nT1[unc(n754)let]* (IV;V), and *dpy-11(e224) mes-4(bn58) V/*D*nT1[unc(n754)let]* (IV;V). See Supporting Information, File S1 for descriptions of alleles.

### Microarray analysis of dissected germlines

A total of 50-70 germlines were dissected from wild-type and mutant (M+Z- generation) young adult hermaphrodites (24 hr after L4 stage) in 1× egg buffer containing 0.1% Tween20, and cut germlines extending from the mitotic tip through meiotic late pachytene were transferred to a tube containing Trizol (Invitrogen) for RNA isolation. The MessageAmp II aRNA Amplification Kit (Ambion) was used to create cDNA, to amplify antisense RNA (aRNA), and to label aRNA with biotinylated UTP. This differs from the labeled amplified RNA preparation method in Tabuchi *et al.* (2011), in which an additional linear amplification step was included. Fragmentation of biotin-labeled aRNA, hybridization to Affymetrix GeneChip *C. elegans* genome arrays, and scanning were performed at the Genomics Core Facility at University of Massachusetts Medical School. Three biological replicates were performed for each strain. Germlines from each genotype were collected and analyzed in parallel to facilitate comparison and are not the same samples used for germline microarray analysis in previous studies (Bender *et al.* 2006; Gaydos *et al.* 2012; Tabuchi *et al.* 2011). We compared our data with these previous microarray data sets and found high reproducibility, despite differences in amplification methods, germline regions harvested, microarray platforms, and *mes-4* alleles used. Correlation coefficients for significantly changed genes in (Tabuchi *et al.* 2011) and our current *lin-54 vs.* wild type are R = 0.92 for autosomal genes and R = 0.76 for X-linked genes; comparing (Gaydos *et al.* 2012) and our current *mes-4 vs.* wild type, the values are R = 0.91 for autosomal genes, R = 0.27 for significantly changed X-linked genes, and R = 0.60 for all X-linked genes.

Statistical analyses were performed with the use of custom scripts and packages in R (http://www.r-project.org) (Ihaka and Gentleman 1996) and Bioconductor (Gentleman *et al.* 2004). The affy package (Gautier *et al.* 2004) was used to quantile normalize the data across replicates (Bolstad *et al.* 2003) and the robust multichip average algorithm was used to obtain probe-set expression values (Bolstad *et al.* 2003). Log_2_-transformed data were used in subsequent analysis and plotting. Statistical analysis for misexpression was performed using the moderated *t*-test from the Bioconductor package limma (Smyth 2004). Statistical significance of misexpression (false discovery rate q) was obtained with the qvalue package (Storey and Tibshirani 2003). Genes with q ≤ 0.05 were called significant for all comparisons. Microarray data were deposited in National Center for Biotechnology Information’s Gene Expression Omnibus and are accessible through GEO series accession number GSE52064.

**Figure 1:**
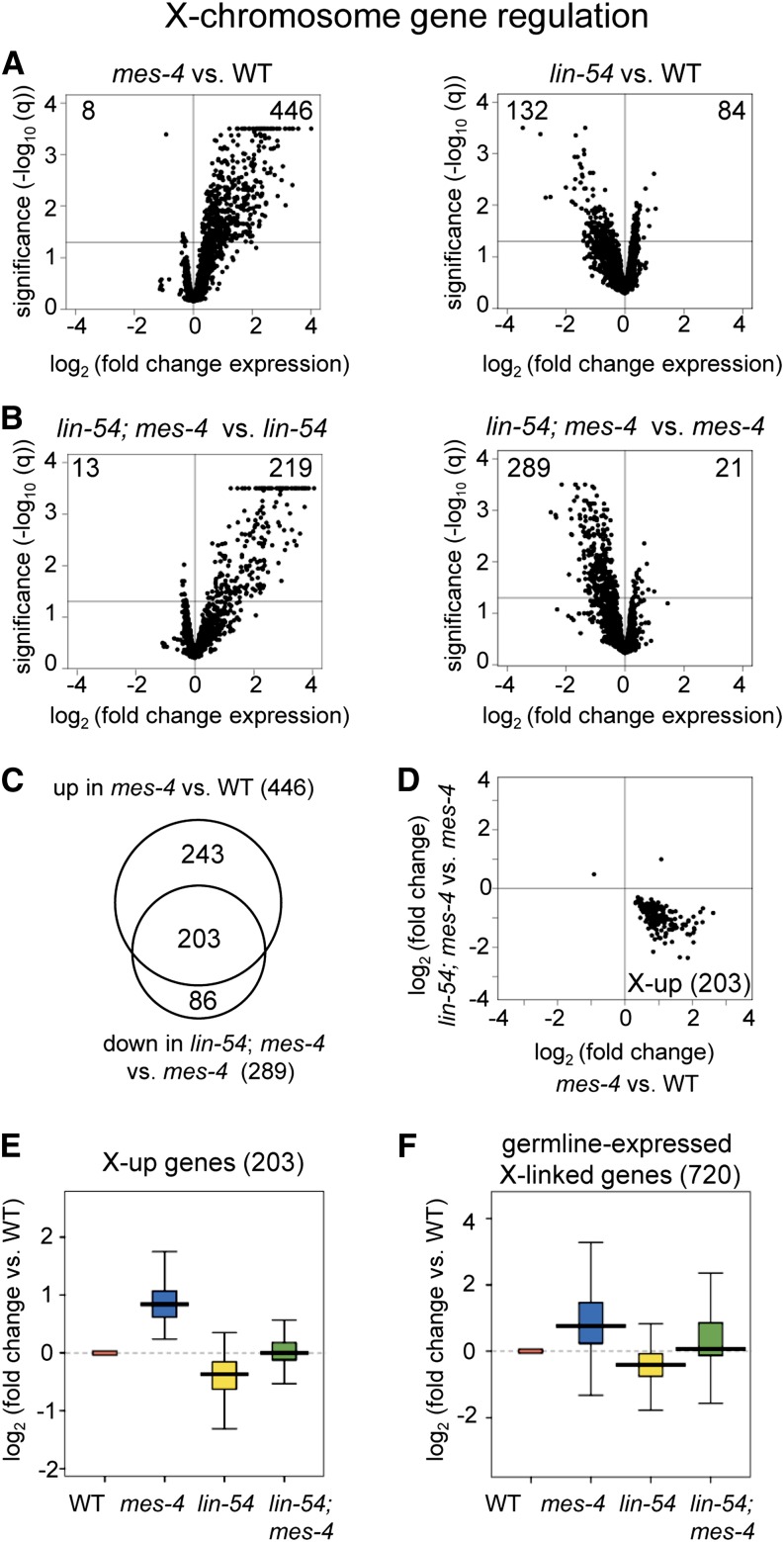
In germ cells, MES-4 and LIN-54 antagonistically regulate X-linked genes, and a double mutant restores more normal X expression. (A-F) Microarray analysis of dissected hermaphrodite germlines showing only X-linked transcripts. (A-B) Volcano plots with x-axis values showing log_2_ of the fold change in transcript level (A) between *mes-4(ok2326) vs.* wild type (WT) and *lin-54(n3423) vs.* WT and (B) between the double mutant *vs. lin-54* or the double mutant *vs. mes-4* single mutant. The y-axis values indicate the statistical significance (−log_10_ q-value) of misexpression of X-linked genes. The gray line marks the significance cutoff of q = 0.05. The numbers of genes significantly up- or down-regulated (q ≤ 0.05) are indicated in the top corners. Genes with −log_10_ q ≥ 3.5 are shown as 3.5. (C) The overlap of X-linked genes significantly up-regulated in *mes-4 vs.* WT and significantly down-regulated in *lin-54*; *mes-4 vs. mes-4* defines 203 genes named “X-up” for their behavior in the *mes-4* mutant (overlap *P* < 10^−99^, hypergeometric test). (D) Scatterplot of X-linked transcripts significantly changed in *mes-4 vs.* WT (q ≤ 0.05) and in the double *vs. mes-4* (q ≤ 0.05). (E-F) Transcript levels (log_2_ fold change) of X-up genes (E) or all germline-expressed X-linked genes (F) in *mes-4(ok2326)* (blue), *lin-54(n3423)* (yellow), and the double mutant (green) relative to WT (red). Each box extends from the 25th to the 75th percentile, with the median indicated by the horizontal line; whiskers extend from the 2.5th to the 97.5th percentile. All differences are statistically significant (Student’s *t*-test, *P* < 0.001), except for WT *vs. lin-54*; *mes-4* in E and F. See also Table S1 and Figure S2 and Figure S3.

**Figure 2:**
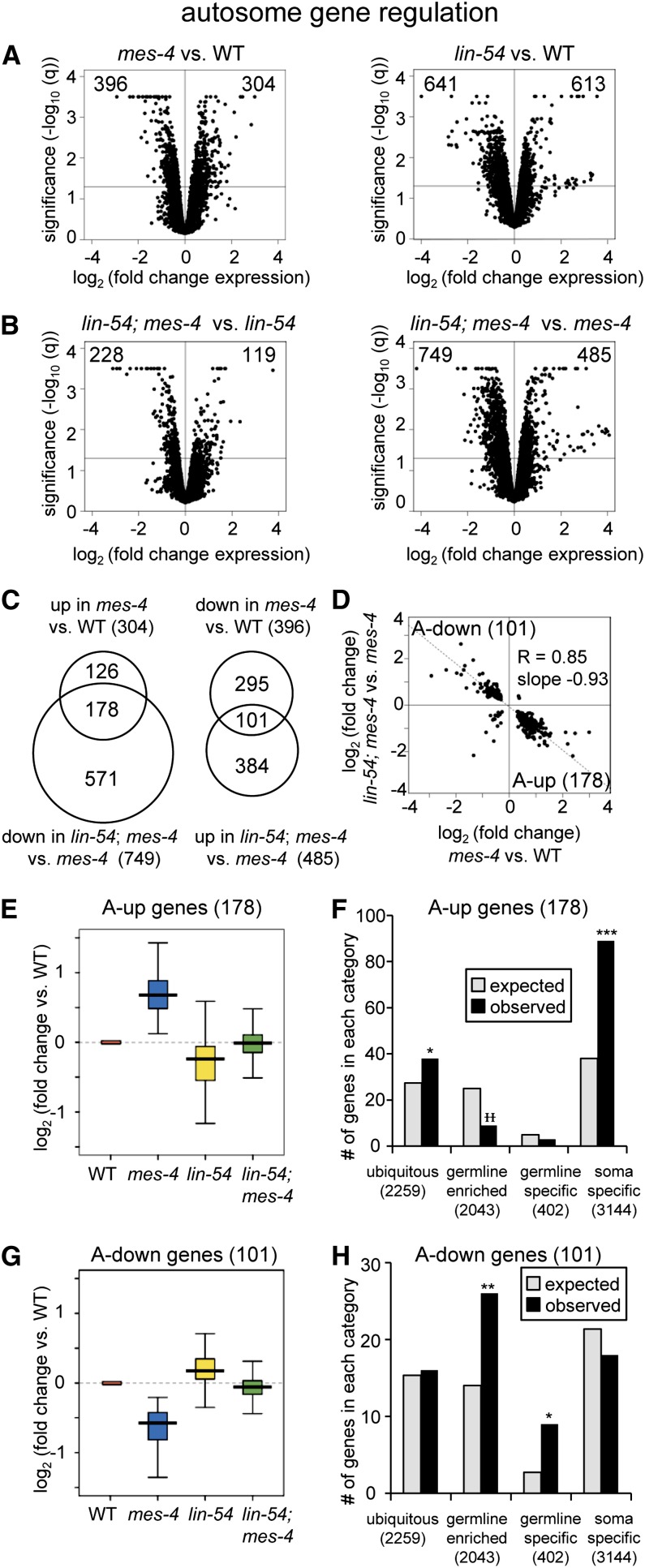
In germ cells, MES-4 and LIN-54 antagonistically regulate two classes of autosomal genes. (A-H) Microarray analysis of dissected hermaphrodite germlines showing only autosomal transcripts. (A and B) Volcano plots with x-axis values showing log_2_ of the fold change in autosomal transcript levels between each single mutant and wild type (WT) or between the double mutant and single mutant, and y-axis values showing statistical significance (−log_10_ q-value) of all autosomal genes on the microarray. The gray line marks the significance cut-off of q = 0.05. The numbers of genes significantly up- or down-regulated (q ≤ 0.05) are in the top corners. Genes with −log_10_ q ≥ 3.5 are shown as 3.5. (C) The overlap of autosomal genes significantly up-regulated in *mes-4 vs.* WT and down-regulated in *lin-54*; *mes-4 vs. mes-4* defines 178 genes, named “A-up” for their behavior in the *mes-4* mutant (overlap *P* < 10^−154^, hypergeometric test). The opposite direction of transcript changes defines 101 “A-down” genes (overlap *P* < 10^−61^). (D) Scatter plot of 296 autosomal genes significantly changed in *mes-4 vs.* WT (q ≤ 0.05) and in the double *vs. mes-4* (q ≤ 0.05); only 17 do not fall into the A-up or A-down categories. This illustrates the mostly opposite directions of transcript fold changes caused by loss of MES-4 and LIN-54. Linear regression analysis yields a slope of -0.93, R = 0.85, indicating strong anti-correlation (*P* < 0.001). (E and G) Transcript levels (log_2_ fold change) of A-up genes (E) or A-down genes (G) in *mes-4(ok2326)* (blue), *lin-54(n3423)* (yellow), and the double mutant (green) relative to WT (red). Each box extends from the 25th to the 75th percentile, with the median indicated by the horizontal line; whiskers extend from the 2.5th to the 97.5th percentiles. All differences are statistically significant (Student’s *t*-test, *P* < 0.001), except for *lin-54*; *mes-4 vs.* WT. (F and H) Expected (gray) and observed (black) numbers of A-up genes (F) or A-down genes (H) in different expression categories defined in (Gaydos *et al.* 2012). Significant enrichment (*) or depletion (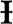) over expected are indicated (*P < 0.05, **P < 0.001, ***P < 10^−10^ by hypergeometric test). See also Table S1, Figure S2, and Figure S3.

For box-and-whisker plots in Figure 1, Figure 2, Figure S1, A and B, and Figure S4, the transcript level of each gene within the set was represented by its normalized probe set log_2_ intensity (or the average of multiple probe sets corresponding to one gene). For the box-and-whisker plot in Figure S1C, log_2_(normalized read depth per transcript + 1) were plotted (data can be accessed at http://intermine.modencode.org/ under accession ID 4006). Log_2_-fold change expression values compared with the wild type were calculated for each gene in each strain. Boxes extend from the 25th to the 75th percentile, with the median indicated by a horizontal line; whiskers extend to the 2.5th and 97.5th percentiles. The Student’s *t*-test was used to calculate statistical significance.

To define expressed genes in Figure 1F, we used the present/marginal/absent calls generated by the mas5calls algorithm in the Bioconductor affy package. We required two or more present calls, or at least one present call and at least one marginal call among three biological replicates. To define expressed genes in Figure S4A, the top 720 highly expressed X-linked genes in wild-type soma (L1 larvae) were selected (Petrella *et al.* 2011).

Except where noted, if multiple probe sets correspond to the same gene annotation, the probe set with the most statistical significance of misexpression was used. The area-proportional Venn diagrams were created using the VennDiagram package, and the statistical significance of overlap was calculated using the hypergeometric test in R.

### Phenotypic analysis of germlines in single and double mutants

**Figure 3:**
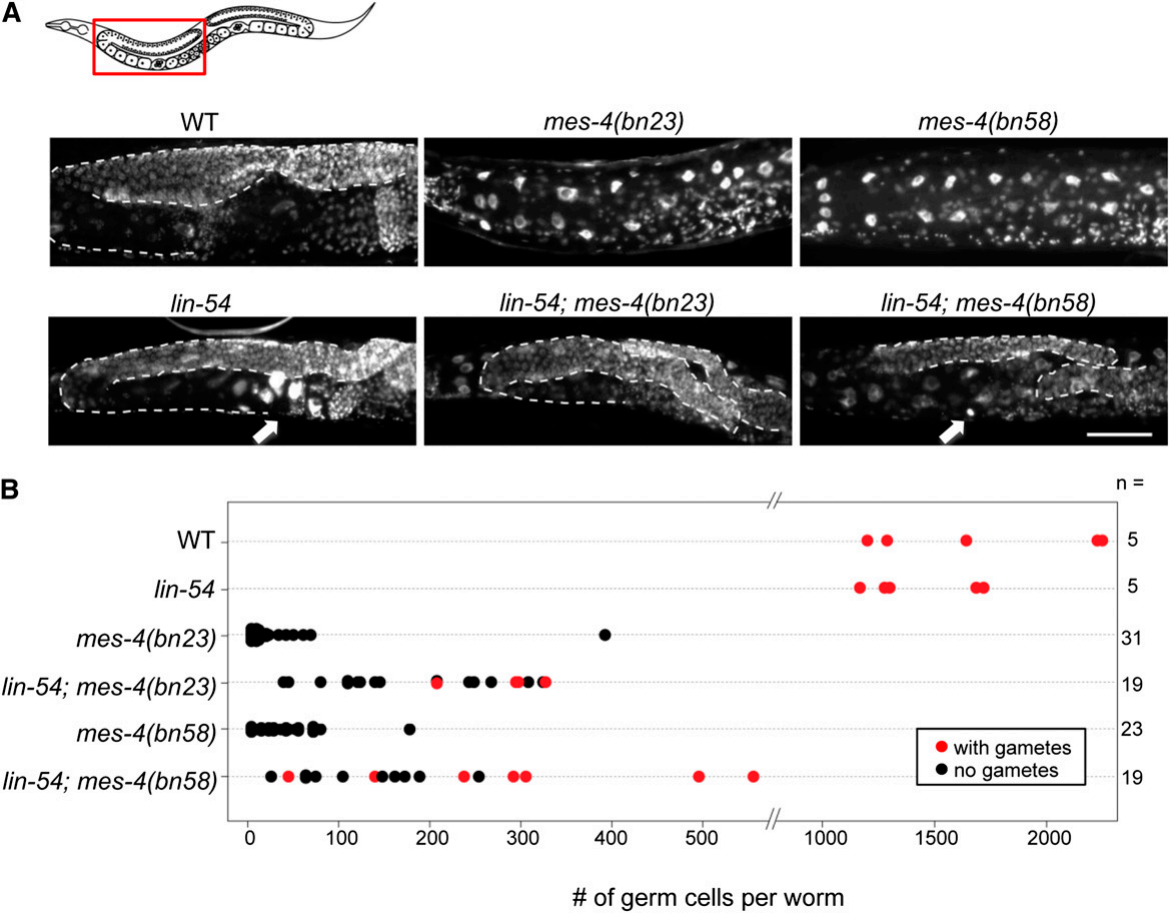
DNA staining of adult hermaphrodites in wild type (WT) and in mutants at the M-Z- generation with the following genotypes: *lin-54(n2990)*, *mes-4(bn23)*, *mes-4(bn58)*, *lin-54(n2990); mes-4(bn23)*, and *lin-54(n2990); mes-4(bn58)*. The images show a region including germ cells in one of the two gonad arms (indicated by the box in the illustration), with the distal end of each gonad arm oriented to the top right and the vulva at the bottom right. Gonad arms are outlined with dashed white lines to show the extent of germline proliferation. The *mes-4(bn23)* or *mes-4(bn58)* worms have no or few germ cells, whereas the double mutants have expanded germlines and sometimes contain gametes. Arrows point to endomitotic oocytes in *lin-54* and *lin-54*; *mes-4(bn58)*. Because the *mes-4(bn23)* and *mes-4(bn58)* alleles are linked to the phenotypic marker *dpy-11(e224)*, this mutant was included as a control; *dpy-11* hermaphrodites contained close-to-normal numbers of germ cells (∼1200) and gametes (not shown). (B) Germ-cell number per worm in each strain. Germ-cell counts were significantly greater in the *lin-54*; *mes-4* double mutants than in the *mes-4* single mutants (Wilcoxon signed-rank test, *P* < 10^−4^). Worms that contained gametes (sperm, oocytes, or endomitotic oocytes) are indicated with red dots. Scale bar = 50 μm.

L4 stage wild-type or homozygous mutants from heterozygous mothers (M+Z- generation) were transferred to plates, and embryos laid within 24 hr were raised to adults (the M-Z- generation) and analyzed. Young adults were fixed with Carnoy’s solution and stained with the DNA dye DAPI. Images were acquired on a Zeiss Axioskop and processed with Image J and Adobe Photoshop. To facilitate quantification of germ cells in wild type, *lin-54(n2990)* single mutant, and *dpy-11(e224)* mutant animals, gonads were dissected and germ cells in one of the two gonad arms were counted and multiplied by two (Figure 3B). For *mes-4* single and *lin-54*; *mes-4* double mutant animals, germ cells in intact worms were counted.

### Chromatin immunoprecipitation (ChIP) data

**Figure 4:**
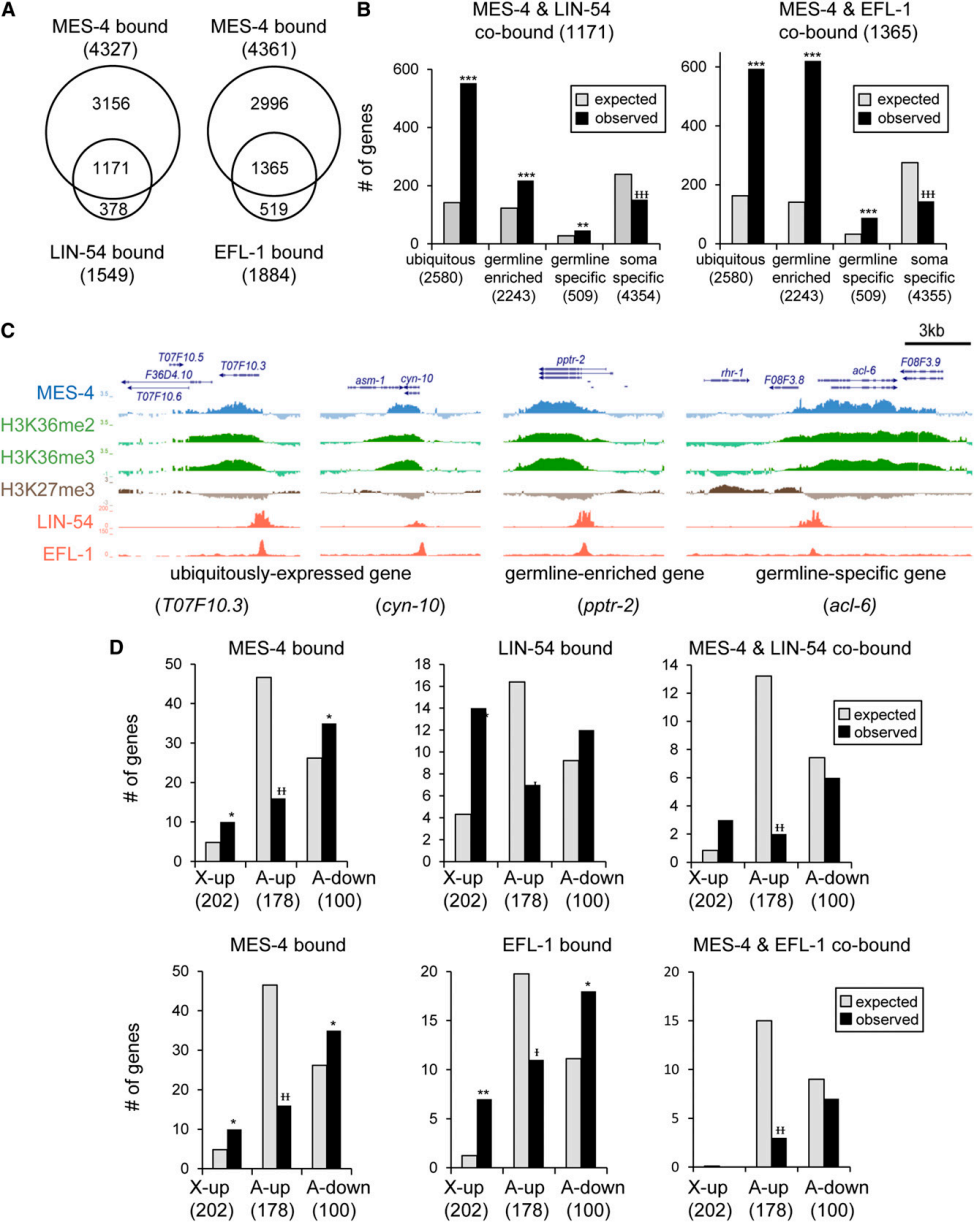
MES-4 and the DRM complex co-bind germline-expressed genes. (A) Overlap of gene bodies bound by MES-4 in embryos (Gaydos *et al.* 2012; Rechtsteiner *et al.* 2010) with gene promoters bound by LIN-54 in mixed-stages (Tabuchi *et al.* 2011) or by EFL-1 in the germline [determined from raw data of (Kudron *et al.* 2013), see *Materials and Methods*]. Overlap significance for both Venn diagrams is *P* < 10^−300^. (B) Expected (gray) and observed (black) numbers of genes co-bound by MES-4 and LIN-54 (left) or by MES-4 and EFL-1 (right) in different gene expression categories defined in (Gaydos *et al.* 2012). (C) View of ChIP binding data at representative genes expressed in the germline, which are enriched for MES-4 and H3K36me2/3 over the gene bodies (blue and green, respectively), depleted for H3K27me3 (brown), and bound by LIN-54 and EFL-1 (red) at the promoters. (D) Expected (gray) and observed (black) numbers of genes bound by MES-4, LIN-54, EFL-1, and both MES-4 and LIN-54 or MES-4 and EFL-1, among X-up, A-up, and A-down genes defined in Figures 1 and 2. Significant enrichment (*) or depletion (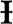) is indicated (* or 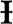 *P* < 0.05, ** or 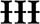 *P* < 0.001, *** or 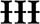 *P* < 10^−10^ by hypergeometric test). See also Figure S5.

ChIP-chip data for MES-4, H3K36me3, and H3K27me3 (in early embryos) can be obtained from GEO under the accession ID GSE38180, and H3K36me2 can be obtained under GSE22717 (Gaydos *et al.* 2012; Rechtsteiner *et al.* 2010). MES-4−bound genes were determined as described in Rechtsteiner *et al.* (2010). ChIP-chip data for LIN-54 (in mixed staged animals) were obtained from GEO under the accession ID GSE28852 (Tabuchi *et al.* 2011); LIN-54−bound genes were determined as described in Tabuchi *et al.* (2011). The LIN-54 genome browser tracks in Figure 4C display the ratio of IP/Input channel intensities. Intensity ratios were scaled to a median absolute deviation of 1, and the median was set to 1. All ChIP-chip data except LIN-54 were obtained on platforms based on genome assembly WS170. LIN-54 was lifted over from WS120 to WS170 using the UCSC Genome Browser liftover utility (http://genome.ucsc.edu/util.html). Ppie-1::EFL-1::GFP ChIP-seq data (EFL-1 expressed under the *pie-1* germline promoter; ChIP performed from young adults) were obtained from GEO under the accession ID GSE30246 (Kudron *et al.* 2013). Raw reads were mapped to WS170 using bowtie with default parameters (Langmead *et al.* 2009). MACS1.4 (Zhang *et al.* 2008) was used to call peaks for the mapped data using a bandwidth parameter of 300 and *P*-value of 10^−5^. Final peak calls for EFL-1 retained only peaks that overlapped in two biological replicates. A gene was called promoter bound by EFL-1 if a peak overlapped at least 200 bp with the region 500 bp upstream from the transcript start site to 500 bp downstream from the transcript start site as obtained from Wormbase.

## Results

### In germ cells, MES-4 and LIN-54 antagonistically regulate X-linked genes, and a double mutant restores more normal X expression

X chromosomes in many species and tissues are subject to special chromosome-wide forms of gene regulation. In *C. elegans* hermaphrodites, gene expression from the two X chromosomes is dampened in somatic and germ cells, but dampening occurs by different mechanisms in the two cell types. In the hermaphrodite soma, a “dosage compensation complex” related to chromosome condensation factors binds the two X chromosomes and down-regulates their expression to equal that of the single X in males (Meyer 2010). In the hermaphrodite germline, different factors including the MES proteins repress X-chromosome gene expression, and the X chromosome produces lower overall transcript levels than an average autosome (Figure S1A, also compare Figure S2, A−C, to Figure S2, D−F) (Bender *et al.* 2006; Gaydos *et al.* 2012; Kelly *et al.* 2002; Reinke *et al.* 2000; Wang *et al.* 2009). In germline tissue, genes with germline expression that reside on the X chromosome exhibit lower expression than those that reside on the autosomes (Figure S1A) (Reinke *et al.* 2004; Reinke *et al.* 2000). In contrast, in somatic tissues, genes with somatic expression that reside on the X chromosome show expression similar to those that reside on autosomes (Figure S1, B and C) (Deng *et al.* 2011; Gupta *et al.* 2006). Perhaps as an evolutionary consequence of germline X-chromosome repression, fewer germline-expressed genes are located on the X chromosome compared with autosomes (Figure S1D) (Piano *et al.* 2000; Reinke *et al.* 2004; Reinke *et al.* 2000).

Previous studies implicated the MES proteins in repressing the X chromosomes in the germline, and the DRM complex in preventing excessive X-chromosome repression, but did not link the two systems in this process (Bender *et al.* 2006; Gaydos *et al.* 2012; Tabuchi *et al.* 2011). In other cellular contexts, such as vulva development, MES-4 and DRM mutants exhibit genetic antagonism (*e.g.*, Cui *et al.* 2006; Petrella *et al.* 2011; Wang *et al.* 2005; Wu *et al.* 2012). We therefore hypothesized that MES-4 and DRM counteract each other to promote proper germline gene expression, in particular by maintaining adequate but not excessive dampening of X-chromosome expression. To test this idea, we performed microarray analysis of germlines dissected from wild-type, *mes-4(ok2326)* null mutant, *lin-54(n3423)* null mutant, and *lin-54(n3423)*; *mes-4(ok2326)* double-mutant adult hermaphrodites. Because LIN-54 and other DRM subunits bind and regulate gene expression similarly in the germline and because LIN-54 is required for DRM complex formation and DNA binding, we presume that results with LIN-54 reflect activities of a germline DRM complex (Chi and Reinke 2006; Harrison *et al.* 2006; Kudron *et al.* 2013; Tabuchi *et al.* 2011).

Consistent with previous findings, we observed that many X-linked genes were up-regulated in *mes-4* mutant germlines (446 genes, false discovery rate q ≤ 0.05 for all expression differences we report, Figure 1A left, Figure S2A, Table S1A), and many were down-regulated in *lin-54* mutant germlines (132 genes, Figure 1A right, Figure S2B, Table S1B). We then asked whether the double mutant would reverse the trend of X-linked gene misregulation in each single mutant. Indeed, the *lin-54*; *mes-4* double mutant compared with the *mes-4* single mutant showed down-regulation of X-linked genes (289 genes, Figure 1B right, Table S1C). Thus, loss of MES-4 derepresses X-linked genes, and additional loss of LIN-54 brings X-linked gene expression back down. We also compared the *lin-54*; *mes-4* double mutant to the *lin-54* single mutant, and found up-regulation of X-linked genes (219 genes, Figure 1B left, Table S1D). Thus, loss of LIN-54 enhances repression of X-linked genes, and additional loss of MES-4 brings X-linked gene expression back up.

We sought to define common X-linked genes antagonistically regulated by LIN-54 and MES-4. Given the genotypes we analyzed, antagonistically regulated genes could be defined in several ways, which are illustrated at the bottom of Figure S3A. We observed that many of the same X-linked genes with increased expression in the *mes-4* single mutant were down-regulated in the double mutant compared with the *mes-4* single mutant (Figure 1, C and D, Figure S3A, overlap significance *P* < 10^−99^). We used this overlap to define 203 X-linked genes antagonistically regulated by MES-4 and LIN-54, which we named “X-up” genes (referring to their behavior in the *mes-4* mutant, Table S1E). Figure 1D shows the 205 genes significantly misregulated in both the *mes-4* single mutant *vs.* wild type (effect of the *mes-4* mutation) and in the *lin-54*; *mes-4* double mutant *vs.* the *mes-4* single mutant (effect of the *lin-54* mutation). Of these 205 genes, 203 (99%) are the antagonistically regulated X-up genes.

Next, we investigated whether removal of both factors would restore wild-type transcript levels. We found that although transcript abundance of X-up genes increased in the *mes-4* mutant compared with wild type (*P* < 0.001) and decreased in the *lin-54* mutant compared with wild type (*P* < 0.001), transcript abundance returned closer to wild-type levels in the double mutant (no significant difference from wild type, Figure 1E, Figure S2C). These results are reminiscent of transcript dose tuning by antagonistic coactivators and corepressors in mammalian stem cells, where depletion of either regulator oppositely skews target transcript levels, and depletion of both regulators restores more wild-type expression (Hu and Wade 2012; Reynolds *et al.* 2013; Yildirim *et al.* 2011). Interestingly, the counteracting activities of MES-4 and LIN-54 extend to the whole-chromosome scale: the average expression of all X-linked genes we defined as expressed in the wild-type germline (720 genes, see *Materials and Methods*) behaved like X-up genes (Figure 1F). Even when the 203 X-up genes are subtracted from these 720 genes, the remaining genes show the same trend (data not shown). Thus, the critical role in X transcript regulation played by each factor is executed by countering the other factor, such that removal of both restores a closer-to-wild type X-chromosome expression level. We re-analyzed published transcript profiles of *mes-4* and DRM mutant somatic tissue (Petrella *et al.* 2011) and found no such X-chromosome-wide transcriptional regulation by MES-4 and DRM (Figure S4A). Together, these results show that in the hermaphrodite germline, but not in the soma, opposing activities of MES-4 and DRM modulate X-chromosome-wide transcript levels to achieve partial X-chromosome repression.

### In germ cells, MES-4 and LIN-54 antagonistically regulate two classes of autosomal genes

In *C. elegans*, most genes required for germline function reside on the autosomes (Figure S1D) (Kamath and Ahringer 2003; Piano *et al.* 2000; Reinke *et al.* 2000, 2004), perhaps because as X-chromosome repression evolved it imposed pressure for genes required for fertility and viability to relocate to autosomes. MES-4 and DRM show autosome-enriched binding (Fong *et al.* 2002; Tabuchi *et al.* 2011), and in germline tissue each up-regulates and down-regulates many autosomal genes in addition to influencing expression of genes on the X (Figure 2, A and B; Figure S2, D and E) (Chi and Reinke 2006; Gaydos *et al.* 2012; Kudron *et al.* 2013; Tabuchi *et al.* 2011). Previously it was not known whether these two transcriptional regulators shared common targets and belonged to a common pathway of germline gene regulation.

Our analysis revealed that MES-4 and LIN-54 antagonistically regulate two groups of autosomal genes, in reciprocal manners. One set of autosomal genes mirrors the trend seen with X-linked genes: up-regulated in the *mes-4* mutant, and down-regulated in the *lin-54*; *mes-4* double mutant compared with the *mes-4* single mutant. We named this set of genes “A-up” for its behavior in the *mes-4* mutant; these genes in wild-type germlines are repressed by MES-4 and their repression is antagonized by LIN-54 (178 genes, Figure 2, C and D, Table S1F, Figure S3B, overlap significance *P* < 10^−154^). The second gene set is also antagonistically regulated by MES-4 and LIN-54, but reciprocally: down-regulated in the *mes-4* mutant and up-regulated in *lin-54*; *mes-4* compared to *mes-4*. We named this gene set “A-down” because of its behavior in the *mes-4* mutant; these genes in wild-type germlines are activated by MES-4, and their activation is antagonized by LIN-54 (101 genes, Figure 2, C and D, Table S1G, Figure S3C, overlap significance *P* < 10^−61^). The scatter plot in Figure 2D compares expression changes of all genes significantly misexpressed both in the *mes-4* single mutant *vs.* wild type (effect of the *mes-4* mutation) and in the double mutant *vs. mes-4* single mutant (effect of the *lin-54* mutation). Of 296 autosomal genes with significantly altered expression in both experiments, 279 (94%) are antagonistically regulated. The line of best fit has a slope of −0.93 and a correlation coefficient R-value of 0.85 (*P* < 0.001), indicating a strong negative correlation between the two mutants and thus similar degrees of gene misregulation but in opposite directions in the two mutants.

For both sets of autosomal genes, we found a restoration of more wild-type expression levels in the absence of both factors. Expression of A-up genes was increased in the *mes-4* mutant compared with the wild type (*P* < 0.001), decreased in the *lin-54* mutant compared with the wild type (*P* < 0.001), and returned to near-normal levels in the *lin-54*; *mes-4* double mutant (no significant difference from wild type, Figure 2E). Reciprocally, expression of A-down genes was decreased in the *mes-4* mutant compared with the wild type (*P* < 0.001), increased in the *lin-54* mutant compared with the wild type (*P* < 0.001), and returned to near-normal levels in the double mutant (no significant difference from wild type, Figure 2G, Figure S2F). These results demonstrate that MES-4 and LIN-54 antagonistically modulate transcription of two sets of autosomal genes, in reciprocal manners, and that removing both factors restores target autosomal gene expression to nearer-to-normal levels.

### MES-4 and DRM and antagonistic regulation of genes expressed in germline or soma

One role of the DRM complex is to prevent the ectopic activation of germline genes in the soma of *C. elegans*, and this function is antagonized by MES-4 and other germline chromatin factors (Cui *et al.* 2006; Petrella *et al.* 2011; Tabuchi *et al.* 2011; Wang *et al.* 2005; Wu *et al.* 2012). Reciprocally, one role of MES-4 is to prevent expression of somatic genes in the *C. elegans* hermaphrodite germline, protecting pluripotent germ cells from differentiating into somatic cell types (Gaydos *et al.* 2012; Patel *et al.* 2012). These findings prompted us to investigate what types of genes (germline-expressed, somatically expressed, or ubiquitously expressed) are antagonistically regulated by MES-4 and DRM in germline tissue.

First we examined X-up genes. The X-up genes are X-linked genes that go up in the *mes-4* mutant and back down in the double mutant, implying that wild-type MES-4 represses these genes and LIN-54 antagonizes their repression. We found that X-up genes include more genes with ubiquitous, germline-enriched, and germline-specific expression than expected by chance (Figure S4B). Consistently, X-up genes show greater transcript levels than all X-linked genes in wild type, because the latter category includes genes not expressed in the germline (Figure S4C). Next, we considered A-up genes, the 178 autosomal genes that go up in the *mes-4* mutant and back down in the double mutant, which we infer are repressed by wild-type MES-4 and whose repression is antagonized by LIN-54. A-up genes include more genes with ubiquitous and soma-specific expression and fewer genes with germline-enriched expression than expected by chance (Figure 2F). Thus, as in a prior study (Gaydos *et al.* 2012), we find that MES-4 represses somatic genes to prevent their ectopic expression in germline tissue, and we now show that LIN-54 antagonizes this activity. Finally, we considered A-down genes, the 101 autosomal genes whose expression goes down in the *mes-4* mutant and back up in the double mutant, implying that wild-type MES-4 activates these genes and LIN-54 antagonizes their activation. A-down genes include more genes with germline-enriched and germline-specific expression than expected by chance (Figure 2H). Consistently, transcript levels from A-down genes in wild-type germline tissue are higher than those from all autosomal genes, since the latter category includes genes not expressed in the germline (Figure S4D). This result supports previous reports that MES-4 activates germline-expressed genes (Gaydos *et al.* 2012), many of which are on autosomes, and we now show that LIN-54 antagonizes this activity.

Together, our findings show that MES-4 and LIN-54 each have both activating and repressing capability, but act oppositely on common target gene sets. LIN-54 antagonizes the repressive role of MES-4 on the X chromosomes (X-up) and on somatic genes (A-up), and antagonizes the activating role of MES-4 on germline genes (A-down). Removal of either factor skews levels of target transcripts, while removal of both factors restores more wild-type expression levels for these transcripts. We propose that together DRM and MES-4 tune gene expression to levels that are appropriate for germ-cell function.

### Loss of LIN-54 suppresses germ-cell defects caused by loss of MES-4

Disruption of either MES-4 or LIN-54 causes germ-cell defects (Capowski *et al.* 1991; Garvin *et al.* 1998; Tabuchi *et al.* 2011), presumably as the result of to gene misexpression (Bender *et al.* 2006; Gaydos *et al.* 2012; Tabuchi *et al.* 2011). *lin-54* mutants have well-proliferated germlines but produce endomitotic oocytes (Figure 3A) (Tabuchi *et al.* 2011). *mes-4* mutants contain drastically stunted germlines due to death of nascent germ cells (Figure 3A) (Capowski *et al.* 1991; Garvin *et al.* 1998). We wondered whether the more normal patterns of gene expression restored to the germlines of *lin-54*; *mes-4* double mutants would restore germ-cell development. We therefore scored germline proliferation in single and double mutants by quantifying the number of germ cells per worm and their ability to produce gametes (allele choices explained in File S1).

Wild-type adult hermaphrodite germlines, which proliferate in two gonad arms, contain ~1500 germ cells per worm and include gametes (Figure 3, A and B). Germlines of homozygous *lin-54(n2990)* mutants from homozygous mothers (the M-Z- generation, which lacks both maternal and zygotic gene product) proliferate similarly well (~1300 germ cells/worm) and produce gametes, although many oocytes are endomitotic with excess DNA (Figure 3, A and B; endomitotic oocyte indicated by arrow). *mes-4(bn23)* and *mes-4(bn58)* M-Z- mutant germlines contain very few germ cells (Figure 3, A and B; median of 8 and 34 germ cells per worm, respectively). The weakest *mes-4* allele, *mes-4(bn58)*, occasionally produces gametes and embryos (Bender *et al.* 2006; Capowski *et al.* 1991; Garvin *et al.* 1998; Rechtsteiner *et al.* 2010). Strikingly, in *lin-54(n2990)*; *mes-4(bn23)* and *lin-54(n2990)*; *mes-4(bn58)* double mutants, the number of germ cells was significantly increased (median of 203 and 169 germ cells per worm, respectively) compared with *mes-4* single mutants (Figure 3, A and B; Wilcoxon signed-rank test *P* < 10^−4^). Moreover, some double mutant animals produced gametes (sperm, oocytes, or endomitotic oocytes) (Figure 3, A and B; endomitotic oocyte indicated by arrow). Despite the improvement in germline proliferation and gamete production, the double mutant did not restore the production of viable progeny. Of 50−100 animals tested per genotype, only 2% of *lin-54*; *mes-4(bn23)* and 1% of *lin-54*; *mes-4(bn58)* were fertile, compared with 0–1% for *mes-4* alone and 68% for *lin-54* alone (data not shown). Together, our results indicate that the germ-cell proliferation and gamete production defects caused by disruption of *mes-4* are suppressed when both *mes-4* and *lin-54* function are disrupted. The improvement in germline formation and function in *lin-54*; *mes-4* double mutants likely reflects the restoration of gene expression patterns and levels closer to those appropriate for germ cells.

### MES-4 and the DRM complex cobind germline-expressed genes

To begin to address how MES-4 and DRM oppositely regulate gene expression, we assessed the overlap between antagonistically regulated genes and genes bound by MES-4, DRM, and key histone modifications. On genes expressed in the germline, MES-4 binds and catalyzes the active mark H3K36me2/3 and repels the repressive mark H3K27me3, helping to keep H3K27me3 concentrated on the X chromosomes and somatic genes (Bender *et al.* 2006; Gaydos *et al.* 2012; Rechtsteiner *et al.* 2010). We therefore analyzed MES-4, DRM, H3K36me3, and H3K27me3, using available ChIP data. To assess germline patterns of MES-4 and histone marks, we analyzed ChIP data from early embryos, which retain germline chromatin signatures (Gaydos *et al.* 2012; Rechtsteiner *et al.* 2010). To assess germline binding of the DRM complex, we analyzed ChIP data for EFL-1(E2F) obtained from adults carrying tagged EFL-1 expressed from a germline-specific promoter (Kudron *et al.* 2013). In addition, we analyzed LIN-54 ChIP data from mixed-stage worms, which contain but are not limited to germline tissue (Tabuchi *et al.* 2011).

Our analyses indicate that MES-4 and DRM cobind many germline-expressed genes. Of the 1549 genes bound by LIN-54 and the 1884 genes bound by germline-specific EFL-1, 1171 and 1365 also are bound by MES-4, respectively (Figure 4A, *P* < 10^−300^ for both Venn diagram overlaps). Accordingly, EFL-1−bound genes are enriched for binding of MES-4 and H3K36me3 in wild-type animals, an enrichment that is lost in MES-4−depleted animals (Figure S5, A and B). Genes cobound by MES-4 and DRM subunits are enriched for genes normally expressed in the germline, and depleted for genes with soma-specific expression (Figure 4B). Figure 4C illustrates representative genes expressed in the germline, with promoters occupied by the DRM subunits LIN-54 and EFL-1, and gene bodies enriched for MES-4 and H3K36me2/3 and depleted for H3K27me3.

Next, we examined MES-4 and DRM binding enrichment on the three categories of antagonistically regulated genes. The A-up genes show significantly fewer MES-4, LIN-54, and EFL-1 binding peaks than expected by chance (Figure 4D). These results imply that A-up genes, which include soma-expressed genes repressed by MES-4 and whose repression is antagonized by DRM (Figure 2, E and F), may be regulated indirectly (also see Gaydos *et al.* 2012). We predicted that in contrast, the A-down category, which includes autosomal genes activated by MES-4 and whose activation is antagonized by DRM, might be directly bound targets, since this category was enriched for germline-expressed genes (Figure 2, G and H) (Gaydos *et al.* 2012), and because genes cobound by MES-4 and DRM tend to be germline-expressed (Figure 4B). We found that A-down genes are statistically significantly enriched for MES-4, H3K36me3, and EFL-1 binding, but not for LIN-54 binding (Figure 4D and Figure S5C), making it difficult to conclude whether these genes are regulated directly by bound MES-4 and DRM. Similarly, X-up genes show some enrichment for MES-4, LIN-54, and EFL-1 binding (Figure 4D), but the numbers of X-up genes bound by these factors is small, making it difficult to infer direct or indirect regulation. We envision two explanations for our observations concerning binding and gene regulation. First, MES-4 and DRM may in fact co-bind and directly regulate germline-expressed genes. Although this would predict strong binding enrichment on A-down genes, this may be difficult to observe because the A-down gene set is small (101 genes), and because the analysis demands intersections between multiple sets of independent microarray and ChIP data, each adding some noise to the analysis. Alternatively, MES-4 and DRM may tune most germline gene expression patterns indirectly. MES-4 and DRM might both bind and antagonistically regulate only one or a few key targets, which are then responsible for the many altered germline gene expression patterns we observe. Or, MES-4 and DRM may influence gene expression at sites distant from where they are bound, for example by altering long-range chromatin organization.

## Discussion

We show that the DRM transcription factor complex and the histone methyltransferase MES-4 regulate common genes, in opposite directions, to tune transcript levels in the *C. elegans* germline. MES-4 promotes, and DRM limits, transcription of germline-expressed genes. In contrast, MES-4 represses and DRM promotes expression of somatic and X-linked genes. Our findings show that conserved transcriptional regulators implicated in development and cancer provide antagonizing activities that together ensure normal germline transcript levels and germline proliferation, and tune chromosome-wide transcript dosage from the X chromosomes.

Here, we place DRM in a common pathway with MES-4, but with opposite action. The current model for MES-4 function is that MES-4 repels H3K27me3, catalyzed by the polycomb repressive complex 2-like MES-2/3/6 complex, from germline-expressed genes, focusing H3K27me3 on somatic and X-linked genes (Bender *et al.* 2004, 2006; Fong *et al.* 2002; Gaydos *et al.* 2012). Because DRM acts oppositely to MES-4, one model is that DRM may encourage H3K27me3 on germline-expressed genes. A potential link between DRM and H3K27me is suggested by the finding that certain *Drosophila* genes require both DRM and H3K27me2 for silencing (Lee *et al.* 2010). Perhaps in *lin-54* mutants, MES-4 activity is unchecked, causing higher H3K36 methylation of germline genes and more effective repulsion of H3K27 methylation; this could lead to greater levels of repressive H3K27me3 on somatic genes and the X chromosomes, causing their observed down-regulation. A second model is that DRM and MES-4 have antagonistic effects on MRG-1, whose homologs bind H3K36me2/3 and recruit histone modifiers, and whose mutant phenotype resembles that of *mes-4* (Takasaki *et al.* 2007). Perhaps MRG-1 binds and reinforces MES-4−mediated H3K36me2/3, and DRM limits MRG-1 at germline-expressed genes. Future tests of these models will require ChIP of candidate proteins and chromatin marks in DRM mutants with the use of techniques that assess patterns in germline tissue.

MES-4 serves critical germline functions, so why use DRM to restrict its activity? We propose that the mutual antagonism of MES-4 and DRM in the germline serves three purposes. First, it ensures tissue-appropriate transcript doses and prevents them from veering toward excessive or insufficient levels that would be detrimental to germline function. Such tuning mechanisms are critical during development, going beyond simple on/off control to produce varied transcriptional outputs that can achieve different developmental consequences (Reynolds *et al.* 2013). Second, DRM/MES-4 antagonism contributes to the specialized transcript dosage regulation of the sex chromosomes. Together, MES-4 and DRM ensure transcript levels from the X chromosomes that are lower, but not excessively lower, than those from an average pair of autosomes. An interesting question for future study is whether MES-4 and DRM act like established X dosage compensation mechanisms to balance X transcript levels between the sexes. Third, DRM/MES-4 antagonism may specify tissue-specific expression programs yet poise target genes for future changes in transcription. In *C. elegans*, chromatin states of pluripotent germ cells are propagated into early embryos, which divide to form primordial germ cells that retain those states, and somatic cells that reprogram those states (Furuhashi *et al.* 2010; Gaydos *et al.* 2012; Petrella *et al.* 2011; Rechtsteiner *et al.* 2010). We speculate that DRM limits MES-4–mediated H3K36me2/3 to make germline-expressed genes amenable to reprogramming and repression during embryonic somatic differentiation. Consistent with this idea, in DRM mutant animals, germline genes are not properly repressed in the soma, a defect that is suppressed in double mutants also lacking germline chromatin regulators like MES-4 (Cui *et al.* 2006; Petrella *et al.* 2011; Wang *et al.* 2005; Wu *et al.* 2012). Similarly, disruption of the *Drosophila* DRM-associated factor L(3)MBT causes germline gene activation in somatic tumors, and tumorigenesis is suppressed in double mutants also lacking germline specifiers (Janic *et al.* 2010). Reciprocally, in the worm germline MES-4 disruption causes ectopic somatic gene expression, which is suppressed in double mutants also lacking DRM (this study and Gaydos *et al.* 2012), and MES-4 disruption allows introduced somatic transcription factors to reprogram germline to somatic fates (Patel *et al.* 2012). Analogous to antagonism between chromatin modifiers and transcription factors on mammalian stem cell pluripotency genes (Hu and Wade 2012; Reynolds *et al.* 2013), the antagonism of MES-4 and DRM may maintain cell fate distinctions while also keeping chromatin states flexibly poised to go down other fate paths upon receiving developmental cues.

## Supplementary Material

Supporting Information
