# Supplementary material for: Opposing Activities of DRM and MES-4 Tune Gene Expression and X-Chromosome Repression in Caenorhabditis elegans Germ Cells
Source: G3 (Bethesda). 2013 Nov 26;4(1):143–53. doi: 10.1534/g3.113.007849 (PMC3887530; doi:10.1534/g3.113.007849)
Supplement: Supporting Information [file supp_4_1_143__index.html]

Opposing Activities of DRM and MES-4 Tune Gene Expression and X-Chromosome Repression in Caenorhabditis elegans Germ Cells — Supporting Information 

# Opposing Activities of DRM and MES-4 Tune Gene Expression and X-Chromosome Repression in *Caenorhabditis elegans* Germ Cells

## Supporting Information for Tabuchi *et al.*, 2014

**Files in this Data Supplement:**

- Supporting Information - Figures S1-S5, File S1, and Table S1 (PDF, 1 MB)
- Figure S1 - Comparison of X chromosome versus average autosome, in terms of gene expression and gene location. (PDF, 486 KB)
- Figure S2 - X and autosomes differ in their transcript levels in wild-type germlines, and in their response to *mes-4* or *lin-54* mutations. (PDF, 841 KB)
- Figure S3 - Comparisons of X-linked and autosomal (A-linked) genes antagonistically regulated by MES-4 and LIN-54. (PDF, 755 KB)
- Figure S4 - Lack of MES-4/DRM antagonism on X-linked genes expressed in soma, and expression characteristics of antagonistically regulated genes. (PDF, 501 KB)
- Figure S5 - Genes bound by germline DRM and A-down genes are enriched for MES-4/H3K36me3 and depleted of H3K27me3. (PDF, 461 KB)
- File S1 - Supporting Materials and Methods (PDF, 434 KB)
- Table S1 - Sets of misregulated genes identified in this study (tabs A-G). (.xlsx, 244 KB)
