# Supplementary material for: Opposing Activities of DRM and MES-4 Tune Gene Expression and X-Chromosome Repression in Caenorhabditis elegans Germ Cells
Source: G3 (Bethesda). 2013 Nov 26;4(1):143–53. doi: 10.1534/g3.113.007849 (PMC3887530; doi:10.1534/g3.113.007849)
Supplement: Supporting Information [file supp_g3.113.007849_007849SI.pdf]

G3 · Genes | Genomes | Genetics

Opposing activities of DRM and MES-4 tune gene expression and X-chromosome repression in *C. elegans* germ cells

Tomoko M. Tabuchi,[*,§] Andreas Rechtsteiner,[§] Susan Strome,[§] Kirsten A. Hagstrom[*]

[*] Department of Molecular Medicine, University of Massachusetts Medical School, Worcester, MA 01605, USA

[§] Department of Molecular, Cell, and Developmental Biology, University of California Santa Cruz, Santa Cruz, CA 95064, USA

DOI: 10.1534/g3.113.007849

Table S1 Sets of misregulated genes identified in this study (tabs A-G).

Available for download as an Excel file at http://www.g3journal.org/lookup/suppl/doi:10.1534/g3.113.007849/-/DC1

Table S1A	446 X-linked genes up in *mes-4(ok2326)* vs. WT
Table S1B	132 X-linked genes down in *lin-54(n3423)* vs. WT
Table S1C	289 X-linked genes down in *lin-54(n3423)*; *mes-4(ok2326)* vs. *mes-4(ok2326)*
Table S1D	219 X-linked genes up in *lin-54(n3423)*; *mes-4(ok2326)* vs. *lin-54(n3423)*
Table S1E	203 X-up genes. X-linked genes up in *mes-4(ok2326)* vs. WT and down in *lin-54(n3423); mes-4(ok2326)* vs. *mes-4(ok2326)*
Table S1F	178 A-up genes. Autosomal genes up in *mes-4(ok2326)* vs. WT and down in *lin-54(n3423); mes-4(ok2326)* vs. *mes-4(ok2326)*
Table S1G	101 A-down genes. Autosomal genes down in *mes-4(ok2326)* vs. WT and up in *lin-54(n3423); mes-4(ok2326)* vs. *mes-4(ok2326)*

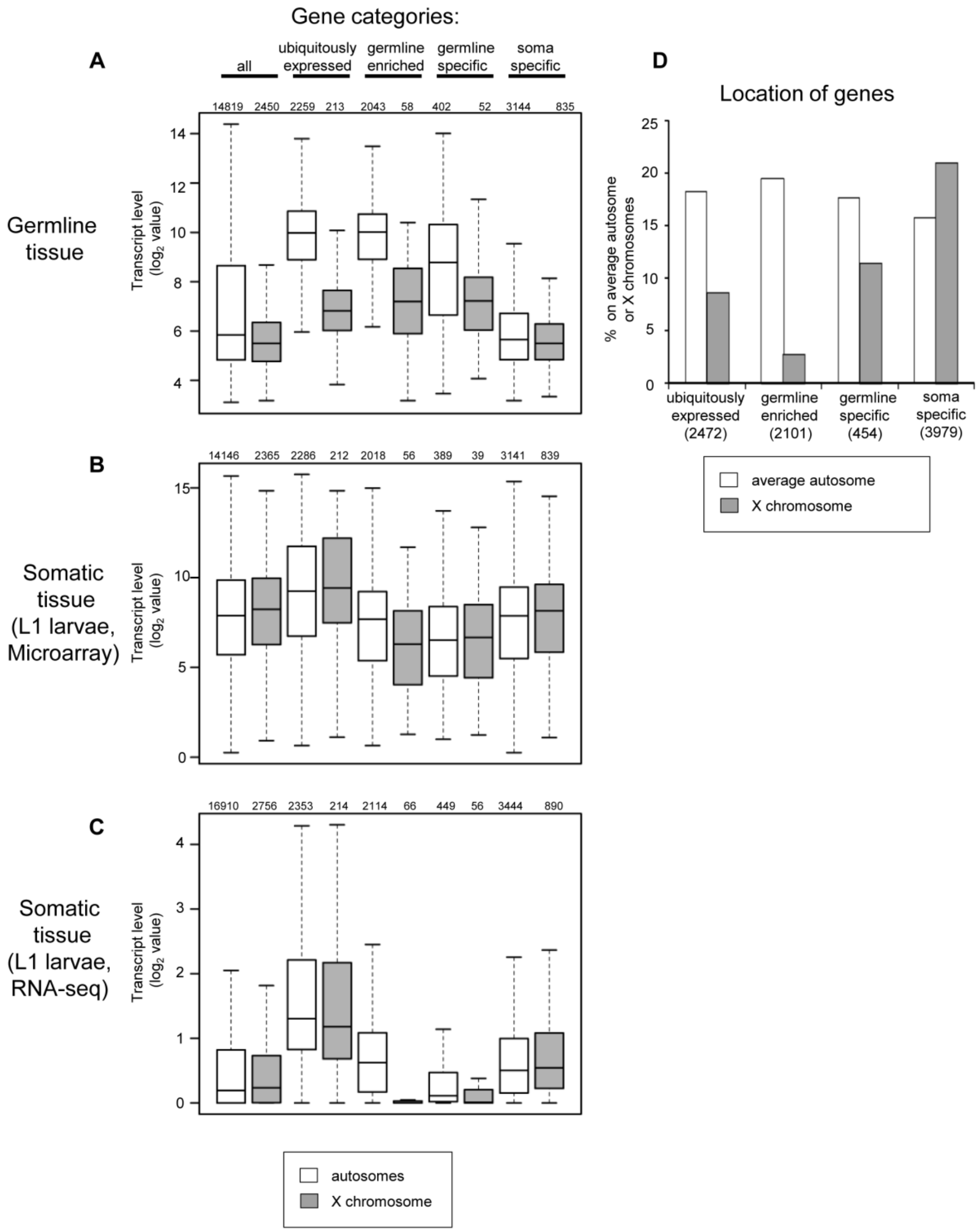

Figure S1 Comparison of X chromosome versus average autosome, in terms of gene expression and gene location. (A-C) Transcript levels of all, ubiquitously-expressed, germline-enriched, germline-specific, and soma-specific genes located on autosomes (white boxes) or on the X chromosome (gray boxes). Transcript levels calculated from (A) dissected adult hermaphrodite germlines (this study, microarray data), (B) L1 larvae which are primarily somatic tissues (microarray data from Petrella *et al.,* 2011, GEO accession ID GSE26824) and (C) L1 larvae (RNA-seq data from Hillier *et al.,* 2009, GEO accession ID 4006). In the germline, gene sets that include genes expressed in germline (all, ubiquitously-expressed, germline-enriched, and germline-specific) exhibit lower transcript levels from genes located on the X compared to autosomes (A). In somatic tissues, gene sets that include genes expressed in the soma (all, ubiquitously-expressed, and soma-specific) exhibit similar transcript levels from genes located on the X and autosomes (B,C). Each box extends from the 25th to the 75th percentile, with the median indicated by the horizontal line; whiskers extend from the 2.5th to the 97.5th percentiles. The numbers on top of the panels indicate the number of genes in each category. (D) Chromosomal location of genes in each expression category (e.g., of 2472 ubiquitously-expressed genes, 2259 are located on the five autosomes, therefore 91% on all five autosomes and 18% on an average autosome). Genes expressed in the germline (ubiquitously-expressed, germline-enriched, and germline-specific) are under-represented on the X chromosome, as described in Reinke *et al.*, 2000.

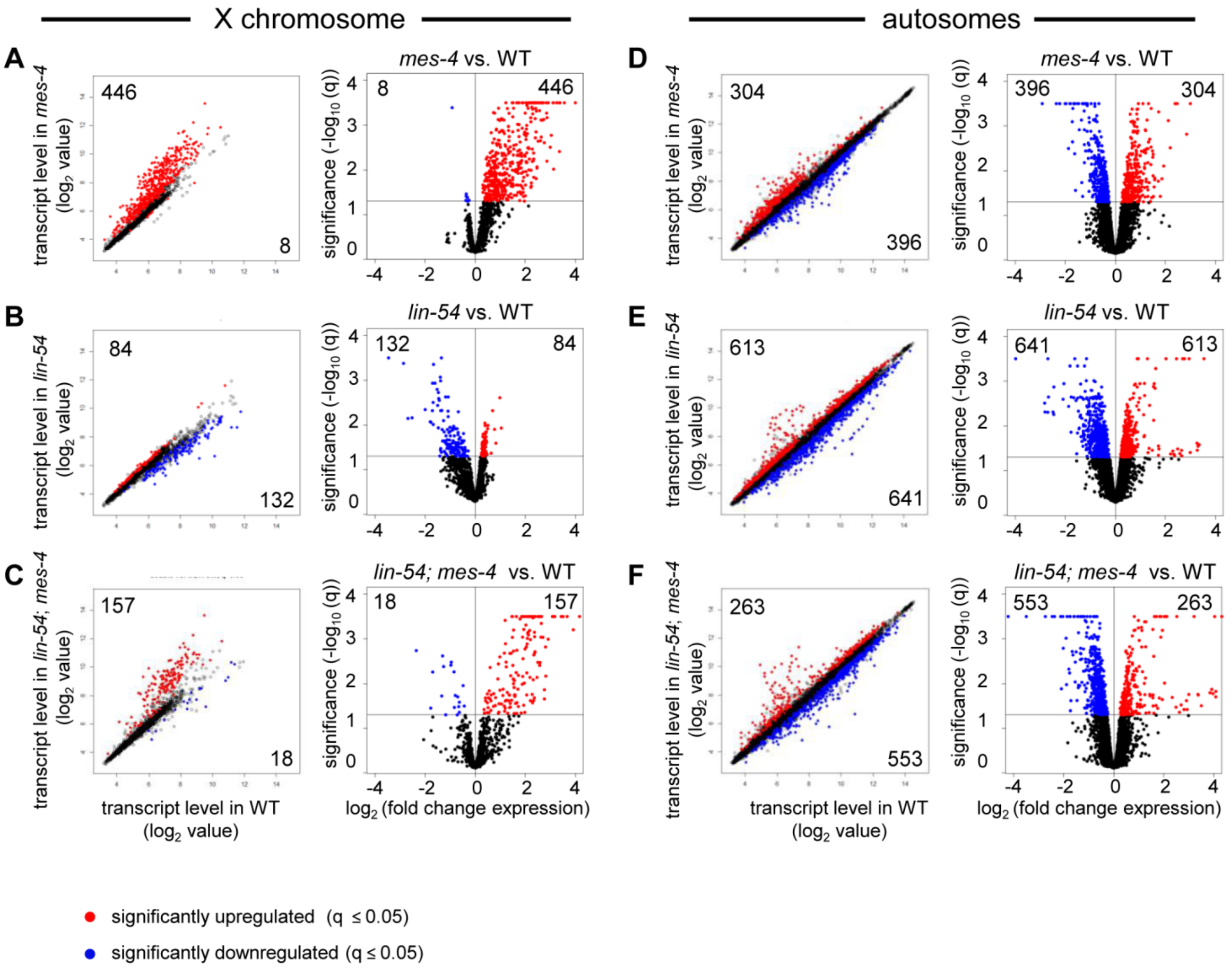

Figure S2 X and autosomes differ in their transcript levels in wild-type germlines, and in their response to *mes-4* or *lin-54* mutations. Scatter plots (left panels) show transcript levels (log_2 intensities) of X-linked genes (A-C) and autosomal genes (D-F). (A, D) *mes-4(ok2326)* (y-axis) vs. WT (x-axis), (B,E) *lin-54(n3423)* vs. WT, and (C, F) *lin-54; mes-4* double mutant vs. WT. Significantly upregulated genes are highlighted in red and downregulated genes in blue ($q \leq 0.05$). In addition to illustrating misregulated genes in mutants, these plots illustrate that in WT the overall transcript levels are lower for the X compared to autosomes. Volcano plots (right panels) show log_2 of the fold change in transcript level on the x-axis and the statistical significance ($-log_{10}$ q-value) on the y-axis. Significantly upregulated genes are highlighted in red and downregulated genes in blue ($q \leq 0.05$). The gray line marks the significance cutoff of q = 0.05. The numbers of genes significantly down- or upregulated are in the top left and right corners. Genes showing significance ($-log_{10}$ q-value) ≥ 3.5 are displayed as 3.5. Volcano plots in A,B,D, and E are also shown in Figures 1 and 2 and are included here for comparison.

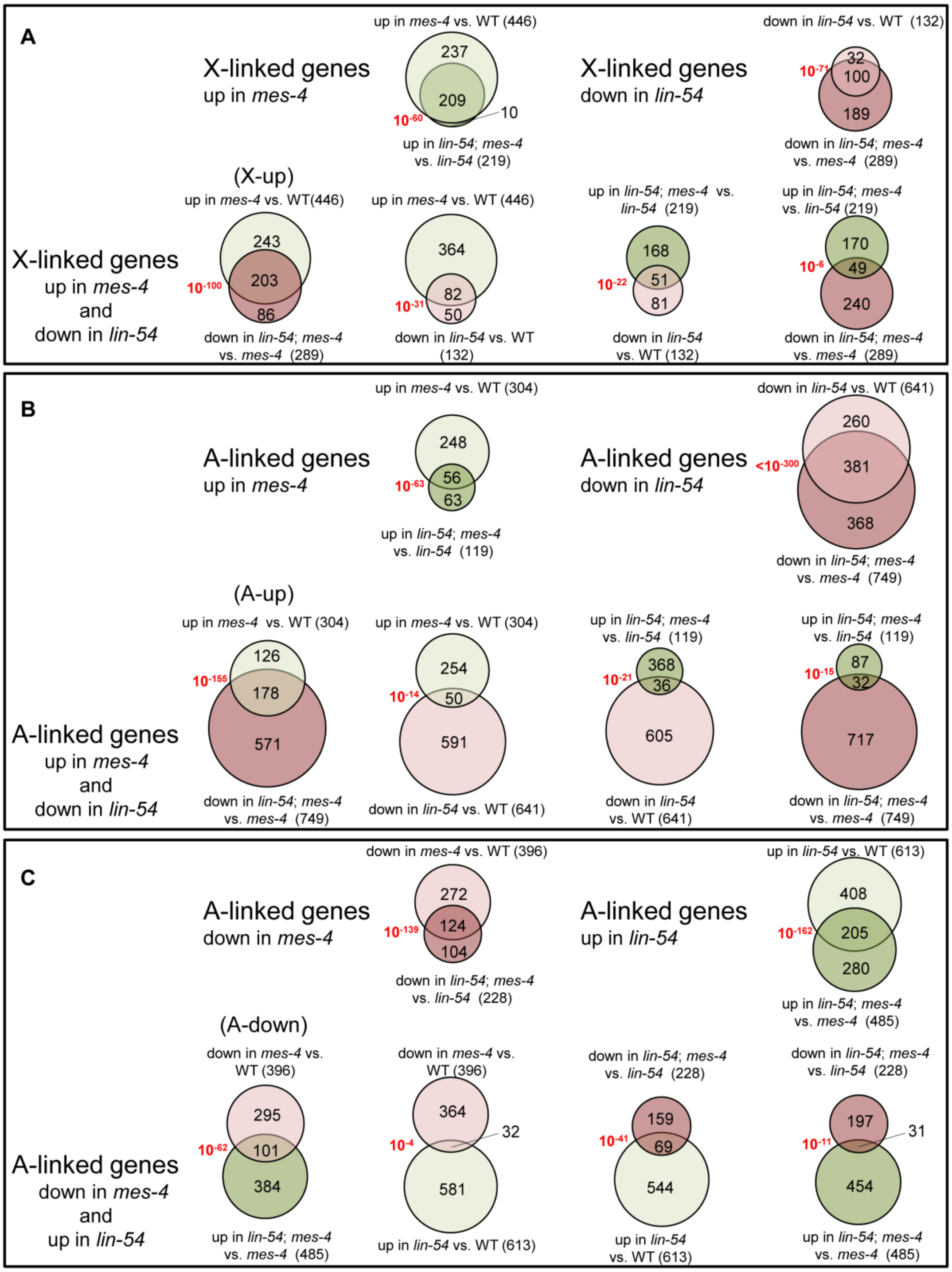

Figure S3 Comparisons of X-linked and autosomal (A-linked) genes antagonistically regulated by MES-4 and LIN-54. Circles indicate gene sets with significantly changed gene expression in indicated genotypes, from our microarray analysis of dissected adult hermaphrodite germlines (See Materials and Methods and Table S1). Upregulated gene sets are shaded green, and downregulated sets are shaded red. (A) X-linked genes whose expression is upregulated in *mes-4(ok2326)* and downregulated in *lin-54(n2423)*. (B) A-linked genes whose expression is upregulated in *mes-4(ok2326)* and downregulated in *lin-54(n2423)*. (C) A-linked genes whose expression is downregulated in *mes-4(ok2326)* and upregulated in *lin-54(n2423)*. The sizes of circles and circle overlap areas are proportional to the number of genes. The statistical significance (p-value) of Venn diagram overlaps is shown by the numbers in red (hypergeometric test). The “X-up”, “A-up”, and “A-down” overlaps we chose to define antagonistically regulated genes for further analysis in the paper are indicated. We elected to focus on those overlaps, instead of the other three options in each category, because they yielded the most significant overlap and the largest number of genes, facilitating further analysis. Also, we reasoned that we gain “sensitivity” by comparing a single mutant (*mes-4*) to the double mutant rather than comparing each single mutant to wild type.

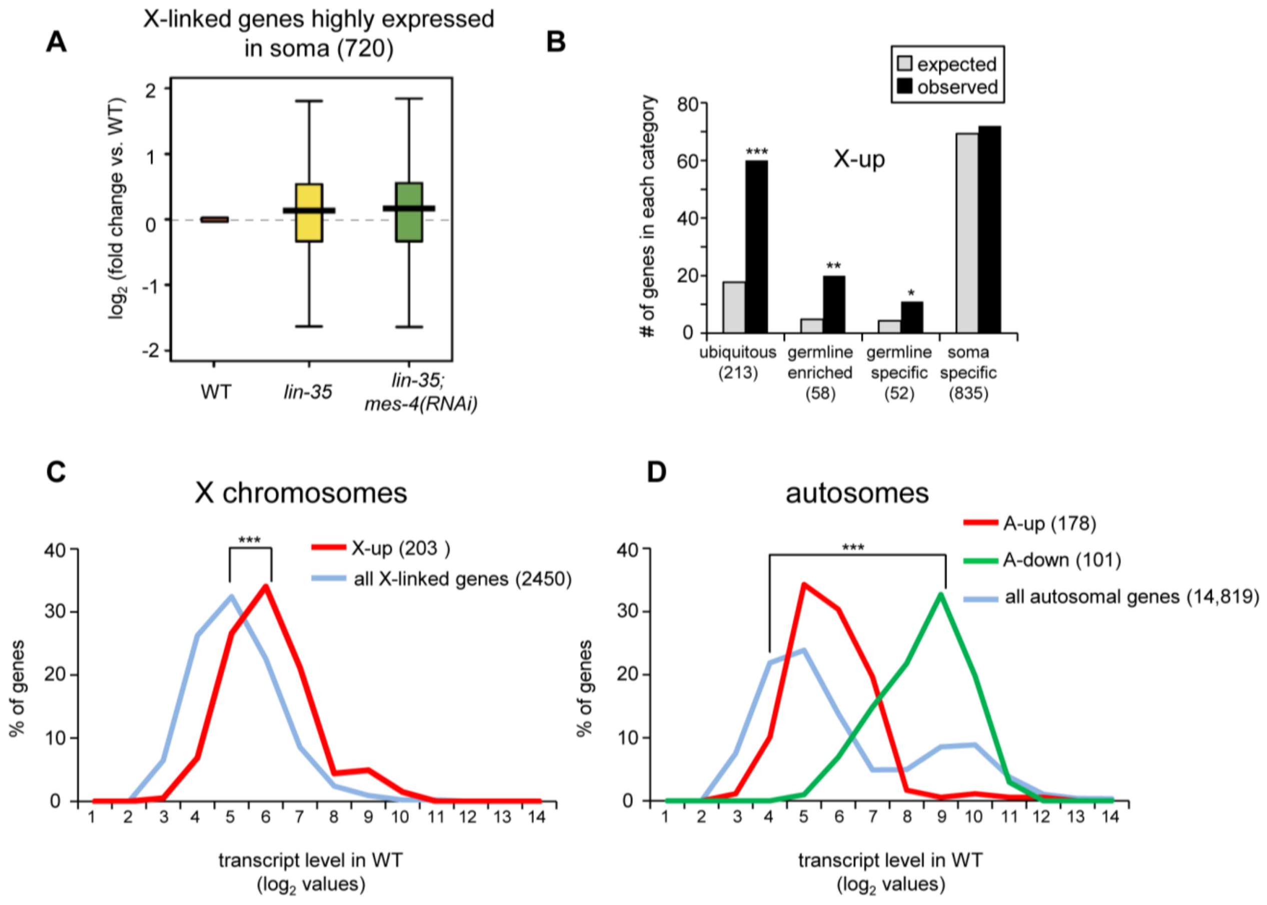

Figure S4 Lack of MES-4/DRM antagonism on X-linked genes expressed in soma, and expression characteristics of antagonistically regulated genes. (A) The transcript level (log_2 fold change) of X-linked genes expressed in L1 larvae (primarily somatic cells) in *lin-35(n745)* (yellow; *lin-35* encodes the *C. elegans* homolog of the Retinoblastoma subunit of DRM) and *lin-35(n745); mes-4(RNAi)* (green) relative to WT (red) (raw data from Petrella *et al.,* 2011). Boxes extend from the 25th to 75th percentile, with the median indicated by a horizontal line; whiskers extend to the 2.5^{th} and 97.5^{th} percentiles. No significant differences were found between the three genotypes, unlike in the germline (see Figure 1F). To be comparable with Figure 1F, the most highly expressed 720 X-linked genes in wild-type L1 larvae were analyzed. (B) Expected (gray) and observed (black) numbers of X-up genes in the indicated expression categories show that X-up genes are enriched for those with ubiquitous and germline expression (*$p < 0.05$, **$p < 0.001$, ***$p < 10^{-10}$ hypergeometric test). (C) Distributions of log_2 transcript levels of X-up genes (red) and all genes on the X chromosome (blue) in WT. % of total genes at each expression level is shown. X-up genes are significantly more highly expressed compared to all X-linked genes (***$p < 10^{-10}$ based on Wilcoxon signed-rank test). (D) Distributions of log_2 transcript levels of A-up genes (red), A-down genes (green), and all autosomal genes (blue) in WT. % of total genes at each expression level is shown. A-down genes have significantly higher expression than all autosomal genes (***$p < 10^{-10}$ based on Wilcoxon signed-rank test), consistent with their enrichment for germline-expressed genes shown in Figure 2H.

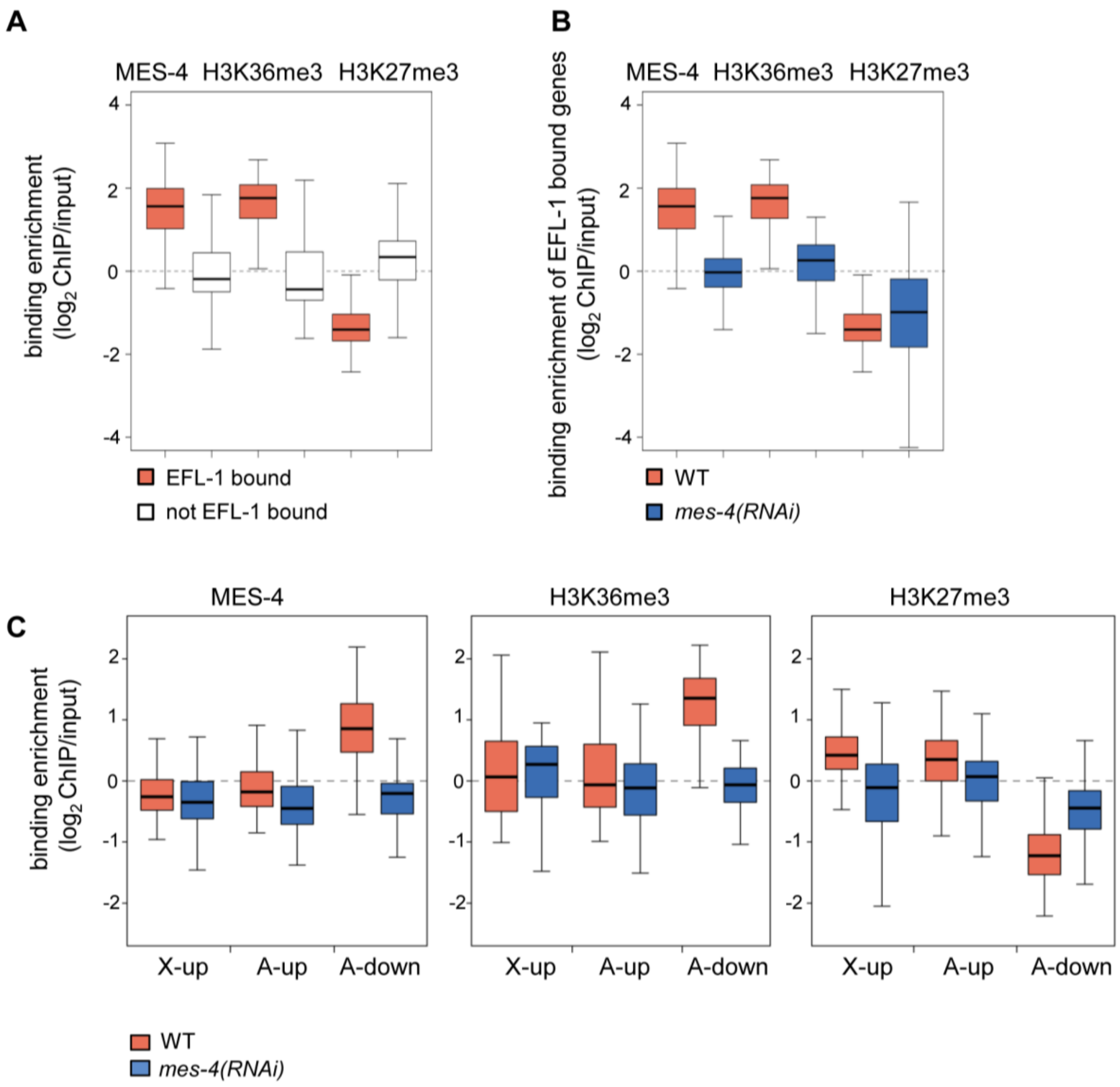

Figure S5 Genes bound by germline DRM and A-down genes are enriched for MES-4/H3K36me3 and depleted of H3K27me3. (A) Binding enrichment or depletion of MES-4, H3K36me3, and H3K27me3 on genes bound by the DRM subunit EFL-1 expressed specifically in the germline (obtained from adults carrying epitope-tagged EFL-1 expressed from a germline-specific promoter, red) and genes not bound by EFL-1 (white) in WT. (B) Binding enrichment or depletion of MES-4, H3K36me3, and H3K27me3 on genes bound by germline EFL-1 in WT (red) and on those same genes in *mes-4(RNAi)* samples (blue). (C) Binding enrichment or depletion of MES-4, H3K36me3, and H3K27me3 on X-up, A-up, and A-down genes in WT (red) and in *mes-4(RNAi)* (blue). Boxes extend from the 25th to 75th percentile, with the median indicated by a horizontal line; whiskers extend to the 2.5th and 97.5th percentiles. All ChIP data analyzed here are available from GEO under accession numbers described in Materials and Methods.

File S1

SUPPORTING MATERIALS AND METHODS

Alleles used for microarray analysis

lin-54(n3423) is a large deletion that removes most of the open reading frame and is a null allele. Adult homozygous *lin-54(n3423)* (M+Z-) animals produce no detectable LIN-54 transcript or protein, and disrupt DRM complex function both by destabilizing other subunit proteins and by preventing DRM subunits from binding to target genes (Harrison *et al.,* 2006; Tabuchi *et al.*, 2011).

mes-4(ok2326) is a deletion that removes much of the SET domain responsible for histone methyltransferase activity, and creates a frameshift in exon 4. In *mes-4(ok2326)* M+Z- homozygous animals, qRT-PCR analysis detected a truncated polyA-tailed *mes-4* transcript (not shown). However, no MES-4 protein or its catalyzed mark H3K36me2 were detected by immunostaining in the M+Z-germline or early M-Z- embryos (not shown); this allele is likely a null. Consistently, homozygous M-Z- adult hermaphrodites contain no or few germ cells.

Alleles used for phenotype analysis

lin-54(n2990) carries a point mutation in the cysteine-rich tesmin/CXC domain, causing disruption of LIN-54 DNA-binding activity and of the ability of DRM subunits to bind and regulate target genes. Compared to the *lin-54(n3423)* null, the *n2990* allele causes similar, but weaker, phenotypes (Harrison *et al.,* 2006; Tabuchi *et al.*, 2011).

mes-4(bn23) is a strong loss-of-function allele caused by a point mutation; animals produce *mes-4* transcript (not shown), but MES-4 protein and H3K36me2/3 are cytologically undetectable (Bender *et al.*, 2006). The *mes-4(bn23)* lesion changes a splice acceptor dinucleotide A$\mathbf{G}_{3130}$ to A$\mathbf{A}_{3130}$ at the 3' end of intron 7. Exon 8 starts with guanine, which acts as a splice acceptor dinucleotide A$\mathbf{A}_{3130}\mathbf{G}$ in the *mes-4(bn23)* mutant, removing the first guanine from exon 8 and causing a frameshift (not shown). Homozygous M-Z- adult hermaphrodites contain no or few germ cells.

mes-4(bn58) is a weak loss-of-function allele caused by a point mutation that results in an amino acid change (R389C). Cytologically, mutant MES-4 protein is detectable but not well associated with chromosomes (Bender *et al.*, 2006). This mutant MES-4 protein has weak histone methyltransferase activity (Bender *et al.*, 2006; Rechtsteiner *et al.*, 2010). This allele causes milder defects in germline proliferation than *mes-4(ok2326) or mes-4(bn23)* (Figure 3).

Phenotype analysis was conducted on different double mutants than the double null mutant subjected to microarray analysis, for reasons explained below. Homozygous *mes-4* mutants from heterozygous mothers (M+Z-) produce germlines with grossly normal appearance but changed gene expression, while their M-Z- progeny have few germ cells and are sterile. We wanted to ask whether germ cell defects of *mes-4* M-Z- mutants are suppressed in a double mutant with *lin-54*. The *lin-54(n3423)* null allele could not be used because it does not produce an M-Z- generation; at the M+Z- generation, germlines appear grossly normal but produce endomitotic oocytes that fail to develop (Harrison *et al.,* 2006; Tabuchi *et al.*, 2011). We therefore used the *lin-54(n2990)* hypomorphic mutant, which was appropriate for our analysis because it produces M-Z- generation worms, shows weaker but similar phenotypes to the null, and makes a DNA-binding defective LIN-54 protein that compromises the ability of DRM subunits to bind and regulate target genes (Tabuchi *et al.*, 2011). We combined the *lin-54* hypomorphic allele with both a strong and a weak *mes-4* allele (*bn23* and *bn58*).

SUPPORTING REFERENCES

Bender, L.B., Suh, J., Carroll, C.R., Fong, Y., Fingerman, I.M., Briggs, S.D., Cao, R., Zhang, Y., Reinke, V., and Strome, S. (2006). MES-4: an autosome-associated histone methyltransferase that participates in silencing the X chromosomes in the C. elegans germ line. Development *133*, 3907-3917.

Harrison, M.M., Ceol, C.J., Lu, X., and Horvitz, H.R. (2006). Some C. elegans class B synthetic multivulva proteins encode a conserved LIN-35 Rb-containing complex distinct from a NuRD-like complex. Proc Natl Acad Sci U S A *103*, 16782-16787.

Hillier, L. W., V. Reinke, P. Green, M. Hirst, M. A. Marra et al., 2009 Massively parallel sequencing of the polyadenylated transcriptome of C. elegans. Genome Res 19: 657-666.

Reinke, V., H. E. Smith, J. Nance, J. Wang, C. Van Doren et al., 2000 A global profile of germline gene expression in C. elegans. Mol Cell 6: 605-616.

Petrella, L.N., Wang, W., Spike, C.A., Rechtsteiner, A., Reinke, V., and Strome, S. (2011). synMuv B proteins antagonize germline fate in the intestine and ensure C. elegans survival. Development *138*, 1069-1079.

Rechtsteiner, A., Ercan, S., Takasaki, T., Phippen, T.M., Egelhofer, T.A., Wang, W., Kimura, H., Lieb, J.D., and Strome, S. (2010). The histone H3K36 methyltransferase MES-4 acts epigenetically to transmit the memory of germline gene expression to progeny. PLoS Genet *6*.

Reinke, V., H. E. Smith, et al. (2000). A global profile of germline gene expression in C. elegans. Mol Cell 6(3): 605-616.

Tabuchi, T.M., Deplancke, B., Osato, N., Zhu, L.J., Barrasa, M.I., Harrison, M.M., Horvitz, H.R., Walhout, A.J., and Hagstrom, K.A. (2011). Chromosome-biased binding and gene regulation by the Caenorhabditis elegans DRM complex. PLoS Genet *7*, e1002074.
