# Supplementary material for: Opposing Activities of DRM and MES-4 Tune Gene Expression and X-Chromosome Repression in Caenorhabditis elegans Germ Cells
Source: G3 (Bethesda). 2013 Nov 26;4(1):143–53. doi: 10.1534/g3.113.007849 (PMC3887530; doi:10.1534/g3.113.007849)
Supplement: Supporting Information [file supp_g3.113.007849_FigureS1.pdf]

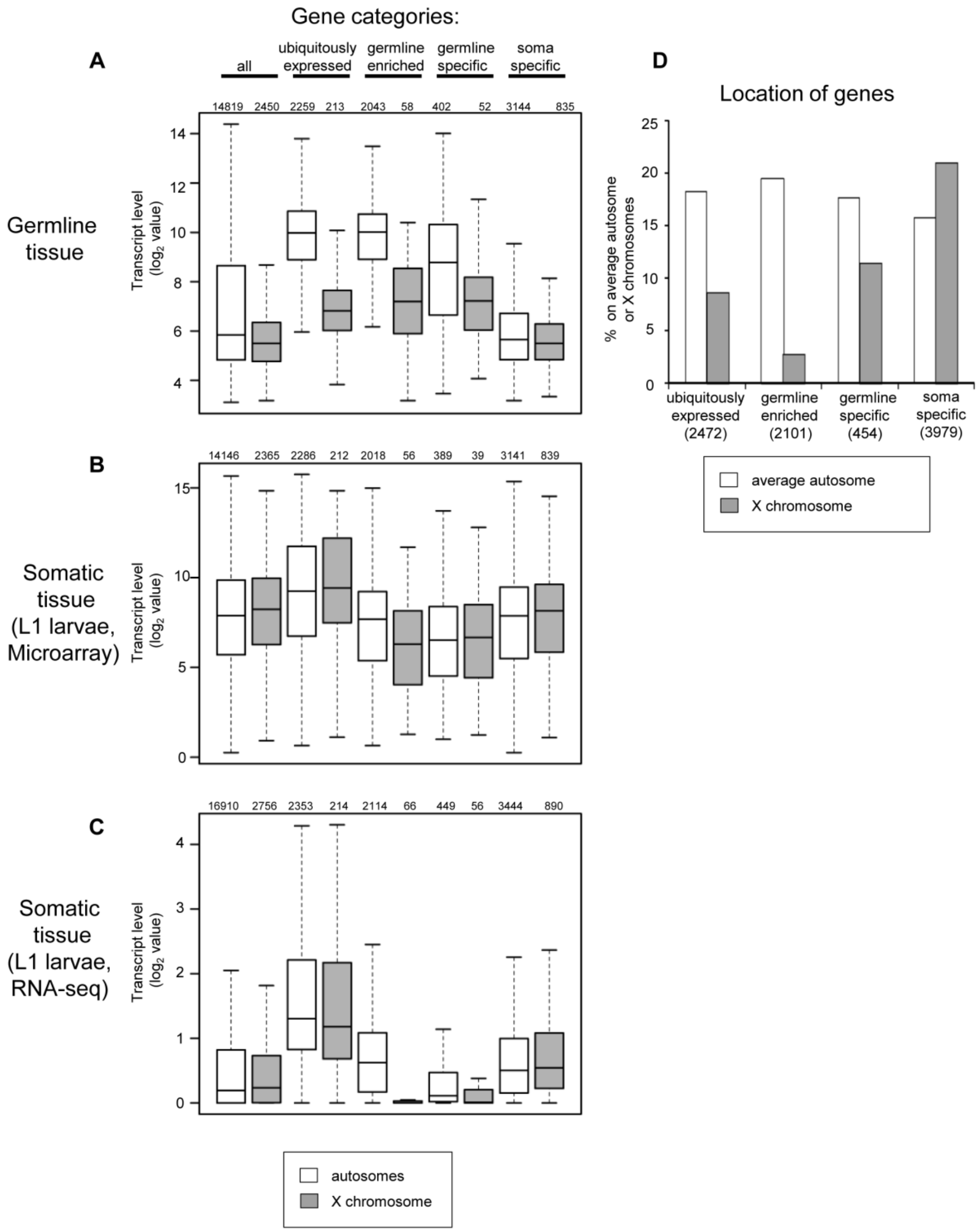

Figure S1 Comparison of X chromosome versus average autosome, in terms of gene expression and gene location. (A-C) Transcript levels of all, ubiquitously-expressed, germline-enriched, germline-specific, and soma-specific genes located on autosomes (white boxes) or on the X chromosome (gray boxes). Transcript levels calculated from (A) dissected adult hermaphrodite germlines (this study, microarray data), (B) L1 larvae which are primarily somatic tissues (microarray data from Petrella *et al.,* 2011, GEO accession ID GSE26824) and (C) L1 larvae (RNA-seq data from Hillier *et al.,* 2009, GEO accession ID 4006). In the germline, gene sets that include genes expressed in germline (all, ubiquitously-expressed, germline-enriched, and germline-specific) exhibit lower transcript levels from genes located on the X compared to autosomes (A). In somatic tissues, gene sets that include genes expressed in the soma (all, ubiquitously-expressed, and soma-specific) exhibit similar transcript levels from genes located on the X and autosomes (B,C). Each box extends from the 25th to the 75th percentile, with the median indicated by the horizontal line; whiskers extend from the 2.5th to the 97.5th percentiles. The numbers on top of the panels indicate the number of genes in each category. (D) Chromosomal location of genes in each expression category (e.g., of 2472 ubiquitously-expressed genes, 2259 are located on the five autosomes, therefore 91% on all five autosomes and 18% on an average autosome). Genes expressed in the germline (ubiquitously-expressed, germline-enriched, and germline-specific) are under-represented on the X chromosome, as described in Reinke *et al.*, 2000.
