# Supplementary material for: Opposing Activities of DRM and MES-4 Tune Gene Expression and X-Chromosome Repression in Caenorhabditis elegans Germ Cells
Source: G3 (Bethesda). 2013 Nov 26;4(1):143–53. doi: 10.1534/g3.113.007849 (PMC3887530; doi:10.1534/g3.113.007849)
Supplement: Supporting Information [file supp_g3.113.007849_FigureS2.pdf]

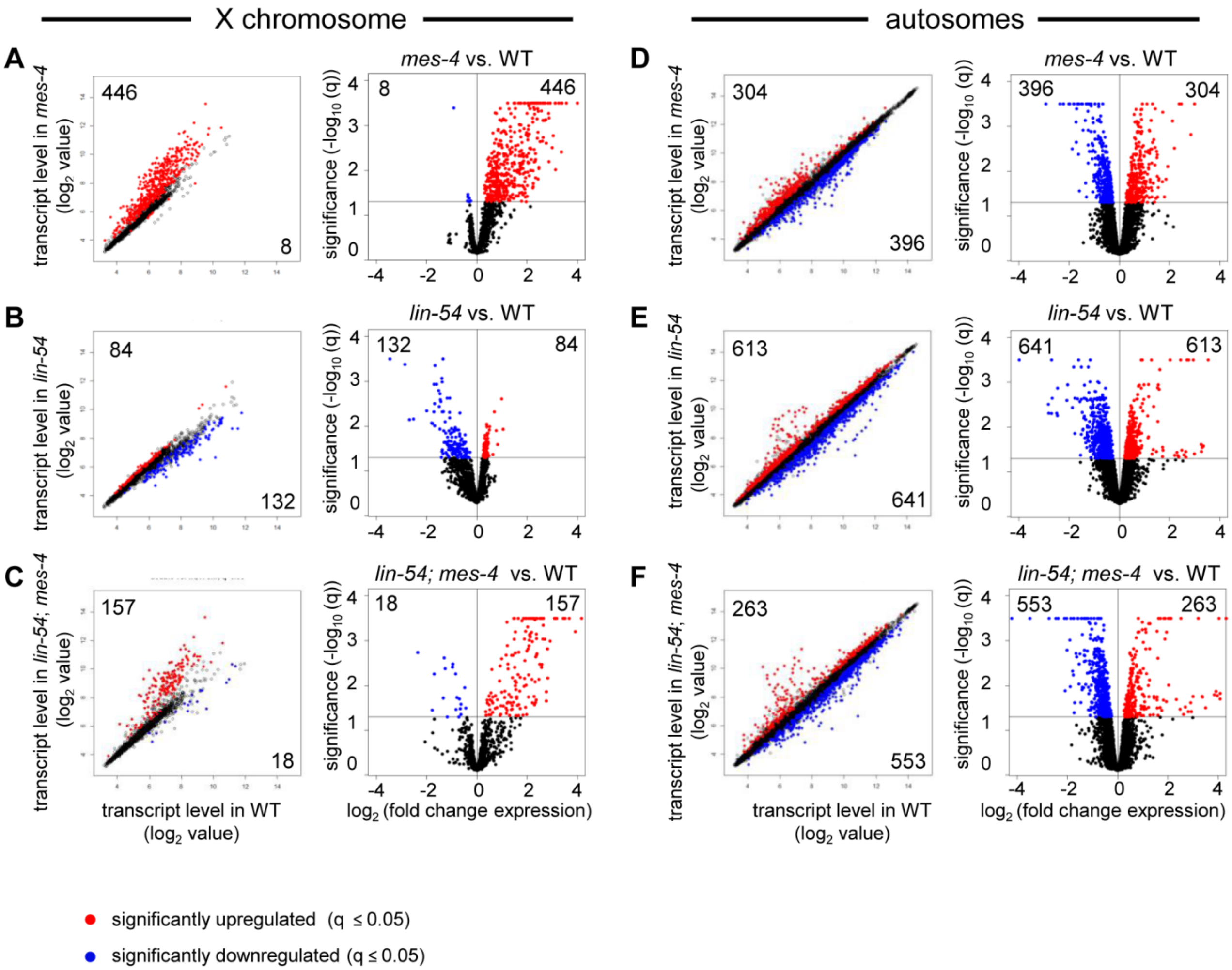

Figure S2 X and autosomes differ in their transcript levels in wild-type germlines, and in their response to *mes-4* or *lin-54* mutations. Scatter plots (left panels) show transcript levels (log_2 intensities) of X-linked genes (A-C) and autosomal genes (D-F). (A, D) *mes-4(ok2326)* (y-axis) vs. WT (x-axis), (B,E) *lin-54(n3423)* vs. WT, and (C, F) *lin-54; mes-4* double mutant vs. WT. Significantly upregulated genes are highlighted in red and downregulated genes in blue ($q \leq 0.05$). In addition to illustrating misregulated genes in mutants, these plots illustrate that in WT the overall transcript levels are lower for the X compared to autosomes. Volcano plots (right panels) show log_2 of the fold change in transcript level on the x-axis and the statistical significance ($-log_{10}$ q-value) on the y-axis. Significantly upregulated genes are highlighted in red and downregulated genes in blue ($q \leq 0.05$). The gray line marks the significance cutoff of q = 0.05. The numbers of genes significantly down- or upregulated are in the top left and right corners. Genes showing significance ($-log_{10}$ q-value) ≥ 3.5 are displayed as 3.5. Volcano plots in A,B,D, and E are also shown in Figures 1 and 2 and are included here for comparison.
