# Supplementary material for: Opposing Activities of DRM and MES-4 Tune Gene Expression and X-Chromosome Repression in Caenorhabditis elegans Germ Cells
Source: G3 (Bethesda). 2013 Nov 26;4(1):143–53. doi: 10.1534/g3.113.007849 (PMC3887530; doi:10.1534/g3.113.007849)
Supplement: Supporting Information [file supp_g3.113.007849_FigureS3.pdf]

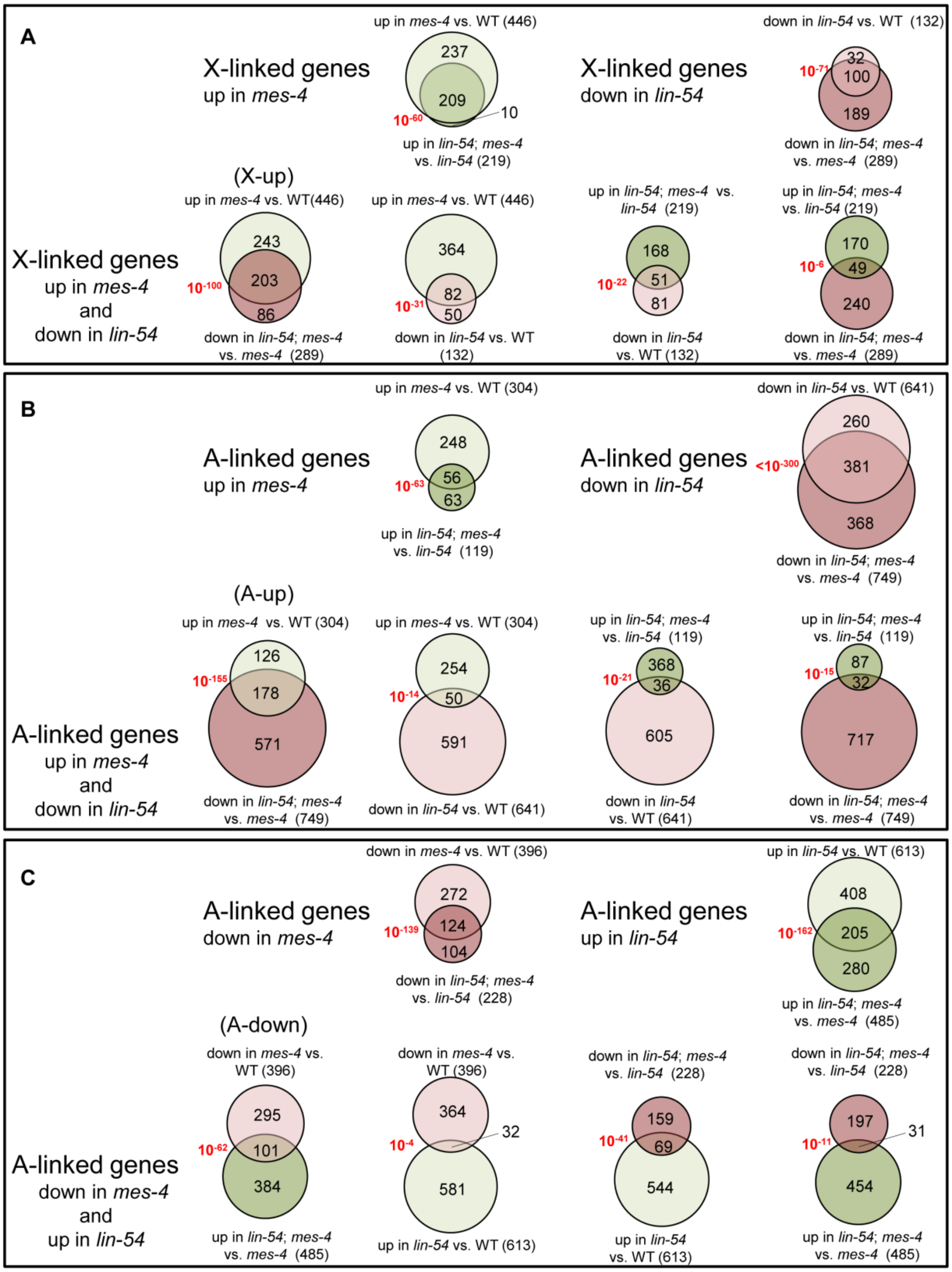

Figure S3 Comparisons of X-linked and autosomal (A-linked) genes antagonistically regulated by MES-4 and LIN-54. Circles indicate gene sets with significantly changed gene expression in indicated genotypes, from our microarray analysis of dissected adult hermaphrodite germlines (See Materials and Methods and Table S1). Upregulated gene sets are shaded green, and downregulated sets are shaded red. (A) X-linked genes whose expression is upregulated in *mes-4(ok2326)* and downregulated in *lin-54(n2423)*. (B) A-linked genes whose expression is upregulated in *mes-4(ok2326)* and downregulated in *lin-54(n2423)*. (C) A-linked genes whose expression is downregulated in *mes-4(ok2326)* and upregulated in *lin-54(n2423)*. The sizes of circles and circle overlap areas are proportional to the number of genes. The statistical significance (p-value) of Venn diagram overlaps is shown by the numbers in red (hypergeometric test). The “X-up”, “A-up”, and “A-down” overlaps we chose to define antagonistically regulated genes for further analysis in the paper are indicated. We elected to focus on those overlaps, instead of the other three options in each category, because they yielded the most significant overlap and the largest number of genes, facilitating further analysis. Also, we reasoned that we gain “sensitivity” by comparing a single mutant (*mes-4*) to the double mutant rather than comparing each single mutant to wild type.
