# Supplementary material for: Opposing Activities of DRM and MES-4 Tune Gene Expression and X-Chromosome Repression in Caenorhabditis elegans Germ Cells
Source: G3 (Bethesda). 2013 Nov 26;4(1):143–53. doi: 10.1534/g3.113.007849 (PMC3887530; doi:10.1534/g3.113.007849)
Supplement: Supporting Information [file supp_g3.113.007849_FigureS4.pdf]

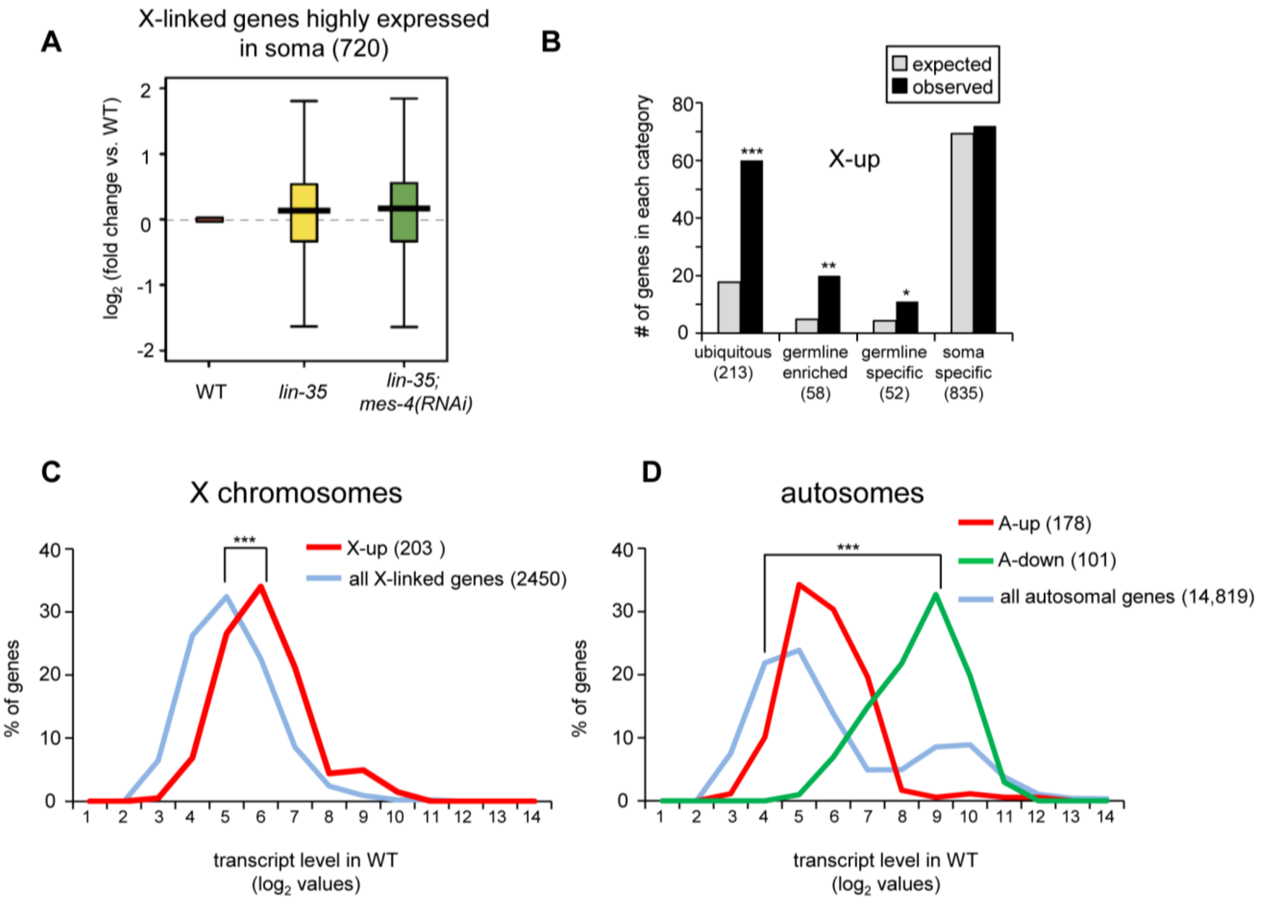

Figure S4 Lack of MES-4/DRM antagonism on X-linked genes expressed in soma, and expression characteristics of antagonistically regulated genes. (A) The transcript level (log_2 fold change) of X-linked genes expressed in L1 larvae (primarily somatic cells) in *lin-35(n745)* (yellow; *lin-35* encodes the *C. elegans* homolog of the Retinoblastoma subunit of DRM) and *lin-35(n745); mes-4(RNAi)* (green) relative to WT (red) (raw data from Petrella *et al.,* 2011). Boxes extend from the 25th to 75th percentile, with the median indicated by a horizontal line; whiskers extend to the 2.5^{th} and 97.5^{th} percentiles. No significant differences were found between the three genotypes, unlike in the germline (see Figure 1F). To be comparable with Figure 1F, the most highly expressed 720 X-linked genes in wild-type L1 larvae were analyzed. (B) Expected (gray) and observed (black) numbers of X-up genes in the indicated expression categories show that X-up genes are enriched for those with ubiquitous and germline expression (*$p < 0.05$, **$p < 0.001$, ***$p < 10^{-10}$ hypergeometric test). (C) Distributions of log_2 transcript levels of X-up genes (red) and all genes on the X chromosome (blue) in WT. % of total genes at each expression level is shown. X-up genes are significantly more highly expressed compared to all X-linked genes (***$p < 10^{-10}$ based on Wilcoxon signed-rank test). (D) Distributions of log_2 transcript levels of A-up genes (red), A-down genes (green), and all autosomal genes (blue) in WT. % of total genes at each expression level is shown. A-down genes have significantly higher expression than all autosomal genes (***$p < 10^{-10}$ based on Wilcoxon signed-rank test), consistent with their enrichment for germline-expressed genes shown in Figure 2H.
