# Supplementary material for: Opposing Activities of DRM and MES-4 Tune Gene Expression and X-Chromosome Repression in Caenorhabditis elegans Germ Cells
Source: G3 (Bethesda). 2013 Nov 26;4(1):143–53. doi: 10.1534/g3.113.007849 (PMC3887530; doi:10.1534/g3.113.007849)
Supplement: Supporting Information [file supp_g3.113.007849_FigureS5.pdf]

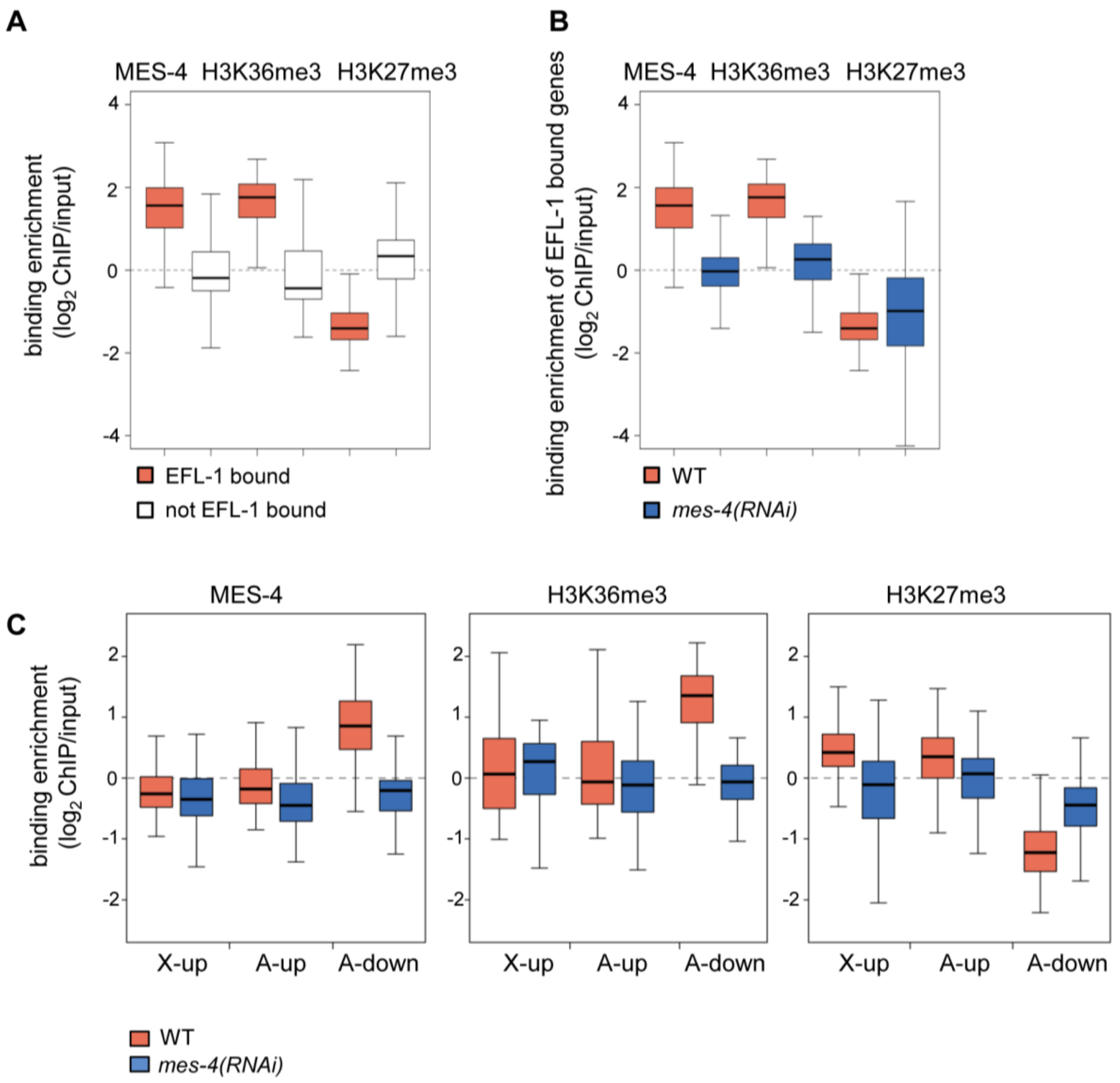

Figure S5 Genes bound by germline DRM and A-down genes are enriched for MES-4/H3K36me3 and depleted of H3K27me3. (A) Binding enrichment or depletion of MES-4, H3K36me3, and H3K27me3 on genes bound by the DRM subunit EFL-1 expressed specifically in the germline (obtained from adults carrying epitope-tagged EFL-1 expressed from a germline-specific promoter, red) and genes not bound by EFL-1 (white) in WT. (B) Binding enrichment or depletion of MES-4, H3K36me3, and H3K27me3 on genes bound by germline EFL-1 in WT (red) and on those same genes in *mes-4(RNAi)* samples (blue). (C) Binding enrichment or depletion of MES-4, H3K36me3, and H3K27me3 on X-up, A-up, and A-down genes in WT (red) and in *mes-4(RNAi)* (blue). Boxes extend from the 25th to 75th percentile, with the median indicated by a horizontal line; whiskers extend to the 2.5th and 97.5th percentiles. All ChIP data analyzed here are available from GEO under accession numbers described in Materials and Methods.
