# Supplementary material for: Opposing Activities of DRM and MES-4 Tune Gene Expression and X-Chromosome Repression in Caenorhabditis elegans Germ Cells
Source: G3 (Bethesda). 2013 Nov 26;4(1):143–53. doi: 10.1534/g3.113.007849 (PMC3887530; doi:10.1534/g3.113.007849)
Supplement: Supporting Information [file supp_g3.113.007849_FileS1.pdf]

File S1

SUPPORTING MATERIALS AND METHODS

Alleles used for microarray analysis

lin-54(n3423) is a large deletion that removes most of the open reading frame and is a null allele. Adult homozygous *lin-54(n3423)* (M+Z-) animals produce no detectable LIN-54 transcript or protein, and disrupt DRM complex function both by destabilizing other subunit proteins and by preventing DRM subunits from binding to target genes (Harrison *et al.,* 2006; Tabuchi *et al.*, 2011).

mes-4(ok2326) is a deletion that removes much of the SET domain responsible for histone methyltransferase activity, and creates a frameshift in exon 4. In *mes-4(ok2326)* M+Z- homozygous animals, qRT-PCR analysis detected a truncated polyA-tailed *mes-4* transcript (not shown). However, no MES-4 protein or its catalyzed mark H3K36me2 were detected by immunostaining in the M+Z-germline or early M-Z- embryos (not shown); this allele is likely a null. Consistently, homozygous M-Z- adult hermaphrodites contain no or few germ cells.

Alleles used for phenotype analysis

lin-54(n2990) carries a point mutation in the cysteine-rich tesmin/CXC domain, causing disruption of LIN-54 DNA-binding activity and of the ability of DRM subunits to bind and regulate target genes. Compared to the *lin-54(n3423)* null, the *n2990* allele causes similar, but weaker, phenotypes (Harrison *et al.,* 2006; Tabuchi *et al.*, 2011).

mes-4(bn23) is a strong loss-of-function allele caused by a point mutation; animals produce *mes-4* transcript (not shown), but MES-4 protein and H3K36me2/3 are cytologically undetectable (Bender *et al.*, 2006). The *mes-4(bn23)* lesion changes a splice acceptor dinucleotide A$\mathbf{G}_{3130}$ to A$\mathbf{A}_{3130}$ at the 3' end of intron 7. Exon 8 starts with guanine, which acts as a splice acceptor dinucleotide A$\mathbf{A}_{3130}\mathbf{G}$ in the *mes-4(bn23)* mutant, removing the first guanine from exon 8 and causing a frameshift (not shown). Homozygous M-Z- adult hermaphrodites contain no or few germ cells.

mes-4(bn58) is a weak loss-of-function allele caused by a point mutation that results in an amino acid change (R389C). Cytologically, mutant MES-4 protein is detectable but not well associated with chromosomes (Bender *et al.*, 2006). This mutant MES-4 protein has weak histone methyltransferase activity (Bender *et al.*, 2006; Rechtsteiner *et al.*, 2010). This allele causes milder defects in germline proliferation than *mes-4(ok2326) or mes-4(bn23)* (Figure 3).

Phenotype analysis was conducted on different double mutants than the double null mutant subjected to microarray analysis, for reasons explained below. Homozygous *mes-4* mutants from heterozygous mothers (M+Z-) produce germlines with grossly normal appearance but changed gene expression, while their M-Z- progeny have few germ cells and are sterile. We wanted to ask whether germ cell defects of *mes-4* M-Z- mutants are suppressed in a double mutant with *lin-54*. The *lin-54(n3423)* null allele could not be used because it does not produce an M-Z- generation; at the M+Z- generation, germlines appear grossly normal but produce endomitotic oocytes that fail to develop (Harrison *et al.,* 2006; Tabuchi *et al.*, 2011). We therefore used the *lin-54(n2990)* hypomorphic mutant, which was appropriate for our analysis because it produces M-Z- generation worms, shows weaker but similar phenotypes to the null, and makes a DNA-binding defective LIN-54 protein that compromises the ability of DRM subunits to bind and regulate target genes (Tabuchi *et al.*, 2011). We combined the *lin-54* hypomorphic allele with both a strong and a weak *mes-4* allele (*bn23* and *bn58*).

SUPPORTING REFERENCES

Bender, L.B., Suh, J., Carroll, C.R., Fong, Y., Fingerman, I.M., Briggs, S.D., Cao, R., Zhang, Y., Reinke, V., and Strome, S. (2006). MES-4: an autosome-associated histone methyltransferase that participates in silencing the X chromosomes in the C. elegans germ line. Development *133*, 3907-3917.

Harrison, M.M., Ceol, C.J., Lu, X., and Horvitz, H.R. (2006). Some C. elegans class B synthetic multivulva proteins encode a conserved LIN-35 Rb-containing complex distinct from a NuRD-like complex. Proc Natl Acad Sci U S A *103*, 16782-16787.

Hillier, L. W., V. Reinke, P. Green, M. Hirst, M. A. Marra et al., 2009 Massively parallel sequencing of the polyadenylated transcriptome of C. elegans. Genome Res 19: 657-666.

Reinke, V., H. E. Smith, J. Nance, J. Wang, C. Van Doren et al., 2000 A global profile of germline gene expression in C. elegans. Mol Cell 6: 605-616.

Petrella, L.N., Wang, W., Spike, C.A., Rechtsteiner, A., Reinke, V., and Strome, S. (2011). synMuv B proteins antagonize germline fate in the intestine and ensure C. elegans survival. Development *138*, 1069-1079.

Rechtsteiner, A., Ercan, S., Takasaki, T., Phippen, T.M., Egelhofer, T.A., Wang, W., Kimura, H., Lieb, J.D., and Strome, S. (2010). The histone H3K36 methyltransferase MES-4 acts epigenetically to transmit the memory of germline gene expression to progeny. PLoS Genet *6*.

Reinke, V., H. E. Smith, et al. (2000). A global profile of germline gene expression in C. elegans. Mol Cell 6(3): 605-616.

Tabuchi, T.M., Deplancke, B., Osato, N., Zhu, L.J., Barrasa, M.I., Harrison, M.M., Horvitz, H.R., Walhout, A.J., and Hagstrom, K.A. (2011). Chromosome-biased binding and gene regulation by the Caenorhabditis elegans DRM complex. PLoS Genet *7*, e1002074.
